# Assessment of the Role of Endothelial and Vascular Smooth Muscle EGFR for Acute Blood Pressure Effects of Angiotensin II and Adrenergic Stimulation in Obese Mice

**DOI:** 10.3390/biomedicines11082241

**Published:** 2023-08-09

**Authors:** Barbara Schreier, Christian Stern, Sindy Rabe, Sigrid Mildenberger, Michael Gekle

**Affiliations:** Julius-Bernstein-Institute of Physiology, Martin-Luther-University Halle-Wittenberg, 06112 Halle, Germany

**Keywords:** epidermal growth factor receptor, blood pressure, metabolic syndrome, vascular smooth muscle cells, endothelial cells, angiotensin II, phenylephrine

## Abstract

(1) Background: Obesity is associated with hypertension because of endocrine dysregulation of the adrenergic and the renin–angiotensin–aldosterone systems. The epidermal growth factor receptor (EGFR) is an important signaling hub in the cardiovascular system. In this study, we investigate the role of smooth muscle cell (VSMC) and endothelial cell (EC) EGFRs for blood pressure homeostasis and acute vascular reactivity in vivo. (2) Methods: Mice with deletion of the EGFR in the respective cell type received either a high-fat (HFD) or standard-fat diet (SFD) for 18 weeks. Intravascular blood pressure was measured via a Millar catheter in anesthetized animals upon vehicle load, angiotensin II (AII) and phenylephrine (PE) stimulation. (3) Results: We confirmed that deletion of the EGFR in VSMCs leads to reduced blood pressure and a most probably compensatory heart rate increase. EC-EGFR and VSMC-EGFR had only a minor impact on volume-load-induced blood pressure changes in lean as well as in obese wild-type animals. Regarding vasoactive substances, EC-EGFR seems to have no importance for angiotensin II action and counteracting HFD-induced prolonged blood pressure increase upon PE stimulation. VSMC-EGFR supports the blood pressure response to adrenergic and angiotensin II stimulation in lean animals. The responsiveness to AII and alpha-adrenergic stimulation was similar in lean and obese animals despite the known enhanced activity of the RAAS and the sympathetic nervous system under a high-fat diet. (4) Conclusions: We demonstrate that EGFRs in VSMCs and to a lesser extent in ECs modulate short-term vascular reactivity to AII, catecholamines and volume load in lean and obese animals.

## 1. Introduction

Metabolic syndrome (MetS) consists of obesity, hyperglycemia, insulin resistance and dyslipidemia, risk factors for cardiovascular diseases and type 2 diabetes (DMT2). Patients with MetS often develop hypertension via modulation of the sympathetic nervous system and the renin–angiotensin–aldosterone system, amongst other pathways [1,2]. The epidermal growth factor receptor (EGFR/ErbB1/Her1) is a tyrosine kinase receptor that has attracted interest in recent years as a signaling hub in various cell types of the cardiovascular system, acting to integrate and mediate some of the cardiovascular effects of angiotensin II (AII) and adrenergic agonists [3].

The EGFR is a member of the ErbB receptor family, including ErbB2, ErbB3 and ErbB4. These receptors form various homo- and heterodimers. Upon ligand binding (e.g., heparin-bound epidermal growth factor (HB-EGF for the EGFR)), the activated dimers modulate cell differentiation, migration and matrix homeostasis [3]. Additionally, the EGFR, most probably via transactivation, contributes to cardiovascular dysfunction and remodeling [4,5]. Systemic application of EGFR inhibitors has been proven to be beneficial for vascular function and vascular wall homeostasis in diabetic animals [6,7,8,9,10,11,12]. Unfortunately, the previous studies could not differentiate between endothelium-EGFR-dependent and smooth-muscle-EGFR-dependent effects. Therefore, mouse models with conditional deletion of the EGFR in the respective cell type were established [13,14]. We demonstrated that while EGFR in vascular smooth muscle cells (VSMCs) contributes significantly to vascular remodeling and renal end-organ damage [14,15], the EGFR in endothelial cells (ECs) is of less importance in mice fed a high-fat diet [12]. Nonetheless, slight differences in vascular contraction could be observed in isolated aortic and mesenteric arteries [14]. Among the factors influenced by EGFR deletion in VSMCs were the basal blood pressure homeostasis, ageing-related vascular changes [13] and pathological AII action in vivo [15]. Furthermore, this model demonstrated the importance of the VSMC-EGFR for obesity-induced vascular and renal damage [16]. We showed that in mice, with deletion of the EGFR in VSMCs, the vascular responsiveness is altered towards different vasoactive substances, and that deletion of the EGFR in VSMCs protects at least in part from endothelial dysfunction [16]. The contribution of the endothelial EGFR to these factors is still unknown.

However, regional heterogeneity in the effect of a high-fat diet (HFD) on the vascular function of conduit arteries has been reported [17]. Therefore, it is of major interest to investigate whether in vitro differences in vascular reactivity on preselected vessels lead to a change in the overall vascular response in the intact cardiovascular system. In this study, we present data on the acute change in blood pressure, which serves as a surrogate parameter for the integrated vascular reactivity in these animals, and we thereby compare animals with or without obesity/DMT2 [14,16].

## 2. Materials and Methods

All mouse experiments were approved by the local government (Landesverwaltungsamt Sachsen-Anhalt, Germany, Az.: 505.6.3-42502-2-1389 MLU_G; Veterinäramt Stadt Halle, Germany) and conducted in accordance with the National Institutes of Health Guide for the Care and Use of Laboratory Animals and the ARRIVE guidelines, as well as considering the 3Rs principle.

### 2.1. Animals

The animal facility provided a constant temperature of 22 ± 2 °C and relative humidity of 30–60%, under a 12/12 h light–dark cycle with ad libitum access to water and standard chow for the mice. Recently, we generated and described an inducible knockout (KO) for EGFR in VSMCs via the Cre/loxP system by mating *Egfr^flox/flox^ C57BL/6* mice (originally provided by M. Sibilia, Institut für Krebsforschung—MedUni Wien, Vienna, Austria) with *Smmhc-Cre^+/−^ C57BL/6N* mice (*B6.FVB-Tg(Myh11-cre/ERT2)1Soff/J*, originally provided by S. Offermanns, Max Planck Institute, Bad Nauheim, Germany) [13,15]. Furthermore, we also generated and described a conditional KO for EGFR in endothelial cells via the Cre/loxP system by mating the aforementioned EGFR^flox/flox^ mice with mice carrying the Cre recombinase under control of the *Tie2* promotor [14]. Genotyping was performed on punched-out ear tissue using PCR as previously described [4,14]. In the iEgfr^Δ/ΔVSMC^ mice, Cre recombinase is located on the Y chromosome; therefore, only male animals were used. Wild-type (WT) mice without LoxP sites but carrying Cre recombinase under control of the respective promotor served as controls in both strains. At 6 weeks of age, tamoxifen (1 mg of 50 μL Miglyol 812/mouse per day for 5 consecutive days; tamoxifen: Sigma Aldrich, St. Louis, MO, USA, Miglyol: pharmacy of the university clinics Halle, Halle, Germany) was injected into the peritoneal cavity to activate the Cre recombinase in VSMCs. At this age, both wild-type and knockout animals were randomly assigned to either the standard-fat diet (SFD) group or high-fat diet (HFD) group. This applied also to the EC-EGFR animals. The HFD (C1090-60 diet, Altromin, Lage, Germany) contained a 60% fat share of energy versus 10% in SFD. Animals were kept on the respective diet for 18 weeks. Assignment to experimental groups was performed randomly by placeholder numbers before information regarding the animals was obtained. During further experimentation, the genotype of the animals was blinded by pseudonymization (assignment of numbers). Owing to the differences in weight gain, the type of diet could not be blinded.

### 2.2. Invasive Measurement of Blood Pressure

Intravasal blood pressure measurements (diastolic, systolic, mean) were taken in anesthetized (80 mg/kg body weight (BW) ketamine and 120 mg/kg BW xylazine, Sigma-Aldrich, St. Louis, MI, USA) mice as described before [13]. The right jugular vein was cannulated for infusion of 2% bovine serum albumin (Sigma-Aldrich, Steinheim, Germany) in Ringer lactate solution (for 1 L: NaCl 5.9 g, KCl 0.3 g, CaCl2 0.22 g, Na-Lactate 2.8 g, all substances were purchased from Sigma-Aldrich, Steinheim, Germany) at 4 μL/g BW/min. A Millar catheter (size 1F, Millar Instruments, Houston, TX, USA) was inserted into the right carotid artery. After a 20 min stabilization period, systolic, diastolic and mean blood pressure were measured (baseline, Power Lab data acquisition systems, Spechbach, Germany) and pulse pressure was calculated with the LabChart7 software (v. 7.3.8; AD Instruments, Spechbach, Germany). To analyze the impact of volume load on blood pressure, a bolus of 50 μL Ringer lactate with a subsequent infusion of 100 μL Ringer lactate solution (within one min, infusion rate 6 mL/h) was infused via the jugular vein catheter, and the blood pressure was analyzed for 10 min (volume load). Subsequently, to analyze the change in blood pressure, phenylephrine (PE, a bolus of 100 μg PE/kg BW; Sigma-Aldrich, Steinheim, Germany) or angiotensin II (a bolus of 1 µg/kg BW; Sigma-Aldrich, Steinheim, Germany) in 50 µL Ringer lactate solution was given. This was followed by an infusion of 100 μL Ringer lactate solution as described above within a minute (infusion rate 6 mL/h). The change in blood pressure was analyzed for a subsequent 20 min period. In a mouse with a mean body weight of 30 g, an infusion of 150 µL would induce a blood volume increase of about 10%.

The impact of VSMC- and EC-EGFR on body weight (Supplementary Figure S1a,b), blood glucose and vascular morphology has been published before [14,16]. As the blood pressure was different under baseline conditions in WT and KO animals, we normalized the change in blood pressure to the blood pressure within the 5 s before the beginning of the infusion to evaluate the vascular reactivity. Therefore, the mean of blood pressure (systolic, diastolic, and mean), heart rate and pulse pressure for every 5 s, and normalized to the beginning of the respective treatment, was calculated separately for every animal. Animals that did not reach the end of the respective treatment were excluded from further analysis. To evaluate the effect of the substance independently from the volume effect, the volume load effect was subtracted from the blood pressure effect after AII or PE infusion.

### 2.3. Statistics

The data are presented as mean ± standard error of mean (SEM). ANOVA followed by post hoc testing or Student’s *t*-test was used, as applicable, according to pre-test data analysis using SigmaPlot 12.5. A *p*-value < 0.05 was considered significant.

## 3. Results

### 3.1. Baseline Blood Pressure

#### 3.1.1. Tie2 Animals

**Figure 1:**
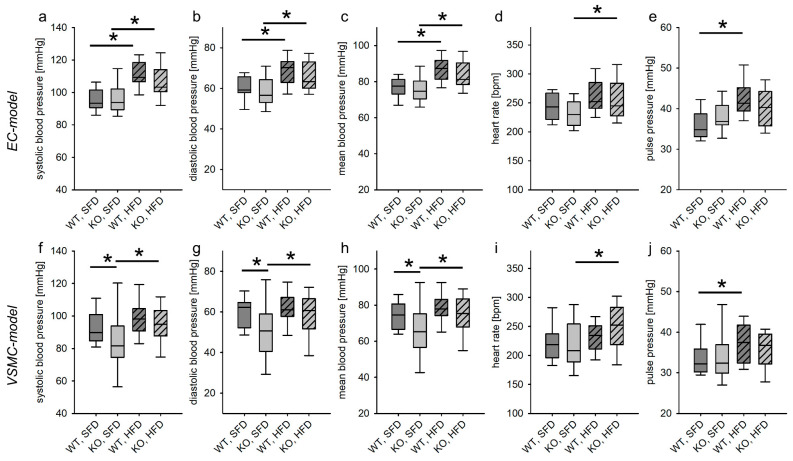
Baseline blood pressure and heart rate in mice with deletion (KO) or without deletion (WT) of the EGFR in endothelial cells (**a**–**e**) or vascular smooth muscle cells (**f**–**j**) after 18 weeks fed with standard-fat diet (SFD) or high-fat diet (HFD). EC model N(WT, SFD) = 22, N(KO, SFD) = 23, N(WT, HFD) = 22, N(KO, HFD) = 24; VSMC model N(WT, SFD) = 26, N(KO, SFD) = 22, N(WT, HFD) = 26, N(KO, HFD) = 30; * *p* ≤ 0.05 vs. respective control.

We implanted Millar catheters in the right carotid arteries of mice fed either SFD or HFD with or without deletion of the EGFR in ECs. Because the animals were anesthetized with ketamine/xylazine, blood pressure and heart rate were reduced in comparison to conscious animals [18]. As reported before, on a smaller cohort [14], neither systolic blood pressure, diastolic blood pressure, mean blood pressure nor heart rate were different between mice with and without EC-EGFR deletion under SFD. As expected, systolic blood pressure, diastolic blood pressure and mean blood pressure were higher in HFD-fed compared to SFD-fed animals but not between the genotypes (Figure 1a–c). Additionally, after 18 weeks of HFD, the heart rate increased (Figure 1d). Pulse pressure increased only in wild-type animals upon HFD feeding (Figure 1e).

Dicrotic notch pressure (pressure at which the aortic valve closes, Supplementary Figure S1c) could not be obtained in all animals. When comparing dicrotic notch pressure in WT and KO animals fed with standard-fat diet, there was no significant difference between the two groups (N(WT, SFD) = 10 animals, N(KO, SFD) = 9 animals). In both genotypes, dicrotic notch pressure increased significantly when animals were fed the high-fat diet (N (WT, HFD) = 8 animals, N (KO, HFD) = 12 animals/group).

In summary, these data indicate that, if at all, the increase in blood pressure is slightly more pronounced if the EGFR in endothelial cells is present. 

#### 3.1.2. SMMHC Animals

As reported before [13], systolic, diastolic and mean blood pressure were significantly lower in KO, SFD animals compared to WT, SFD animals. HFD led to an increase in systolic as well as diastolic and mean blood pressure in KO animals, resulting in a similar systolic, diastolic and mean blood pressure for both genotypes in the end (Figure 1f–j). Heart rate was not different between the genotypes fed the SFD. In KO animals alone, the HFD induced a slight but significant increase in heart rate (Figure 1i). There was no difference in pulse pressure between WT, SFD and KO, SFD animals (Figure 1j). Upon HFD feeding, the pulse pressure increased in WT animals, but not in KO animals.

There was no difference in the level of the dicrotic notch pressure between the genotypes. While the dicrotic notch pressure was not altered in WT animals fed the HFD, it increased in KO animals upon HFD feeding (Supplementary Figure S1d). Together with the increase in diastolic and mean blood pressure, this indicates an increased peripheral resistance in KO animals due to the HFD.

### 3.2. Blood Pressure Response to Volume Load

#### 3.2.1. Tie2 Animals

**Figure 2:**
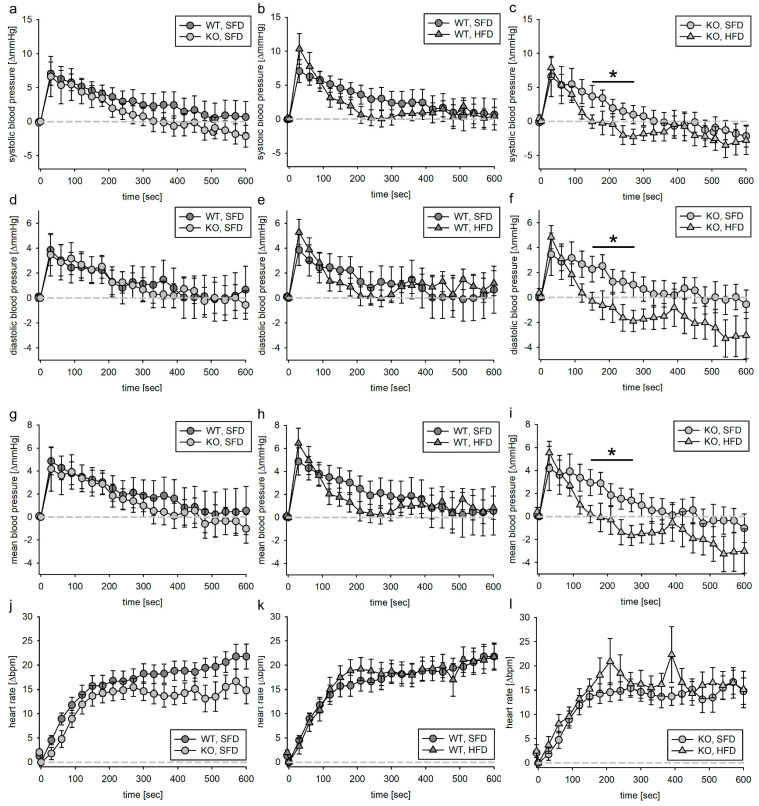
Systolic (**a**–**c**), diastolic (**d**–**f**) and mean (**g**–**i**) blood pressure as well as heart rate (**j**–**l**) response to volume load in the EC model. Vehicle solution was infused in the jugular vein for 1 min and blood pressure as well as heart rate were normalized to the respective value within the 5 s before start of infusion. N (WT, SFD) = 20, N (KO, SFD) = 19, N (WT, HFD) = 21, N (KO, HFD) = 24; data given as mean ± SEM; * *p* ≤ 0.05 vs. respective control.

Volume load alone induced modest changes in blood pressure. The systolic, diastolic and mean blood pressure increased about 7–10 mmHg and returned to initial values within 600 s of analysis in WT as well as in KO animals (Figure 2a,d,g). Of note, the decrease in blood pressure was faster in HFD animals than in SFD animals. Furthermore, KO, HFD animals overcompensated for the volume-load-induced blood pressure increase by reducing the mean blood pressure below the initial volumes (Figure 2a,c,e,f,h,i). Altogether, the changes due to volume load were very low and overinterpretation should be avoided.

As expected, the heart rate of the animals increased with the volume load (Figure 2j), with changes that were indistinguishable between WT and KO as well as HFD and SFD animals (Figure 2k,l).

#### 3.2.2. SMMHC Animals

In the same way as for the Tie2 animals, we also administered Ringer solution to the SMMHC animals. During the infusion or the 10 min observation period, 16 of the 104 animals died. We performed necropsies on all animals included in the experiments; among the animals that died before the end of the experiment, several animals had free blood in the thoracic or abdominal cavity.

**Figure 3:**
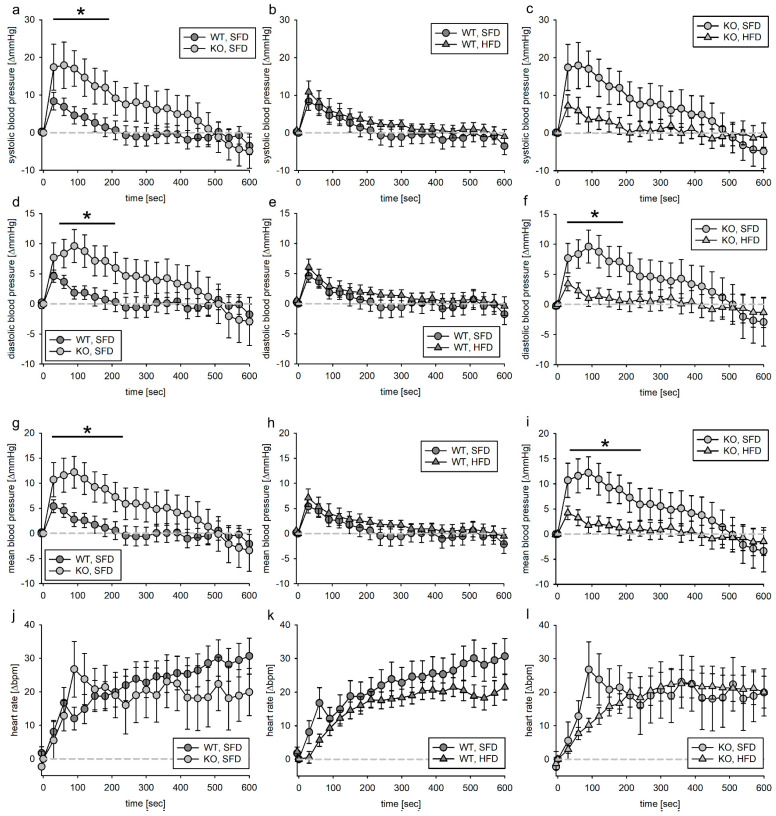
Systolic (**a**–**c**), diastolic (**d**–**f**) and mean (**g**–**i**) blood pressure as well as heart rate (**j**–**l**) response to volume load in the VSMC model. Vehicle solution was infused in the jugular vein for 1 min and blood pressure as well as heart rate were normalized to the respective value within the 5 s before start of infusion. N (WT, SFD) = 25, N (KO, SFD) = 15, N (WT, HFD) = 24, N (KO, HFD) = 17; data given as mean ± SEM; * *p* ≤ 0.05 vs. respective control.

The blood pressure increase in VSMC-EGFR-WT animals fed the SFD was comparable to EC-EGFR-WT animals and returned to initial values within 600 s after the start of the infusion (Figure 3a,d,g). In the corresponding VSMC-EGFR-KO animals, the blood pressure increase upon the volume load was significantly higher. In WT animals, the HFD did not alter the blood pressure response to the volume load (Figure 3b,e,h). In KO animals, the initial blood pressure response was lower than in HFD animals regarding diastolic and mean blood pressure (Figure 3c,f,i). The change in blood pressure response upon volume loading led to an increase in the area under the curve when comparing KO, SFD animals to WT, SFD or KO, HFD, thus indicating an increased blood pressure burden already under the volume load (Supplementary Figure S2). The heart rate increased in all groups to a similar extent upon vehicle infusion. As the changes upon volume load were very low, the impact was probably also low.

### 3.3. Blood Pressure Response to Acute Phenylephrine Infusion

#### 3.3.1. Tie2 Animals

**Figure 4:**
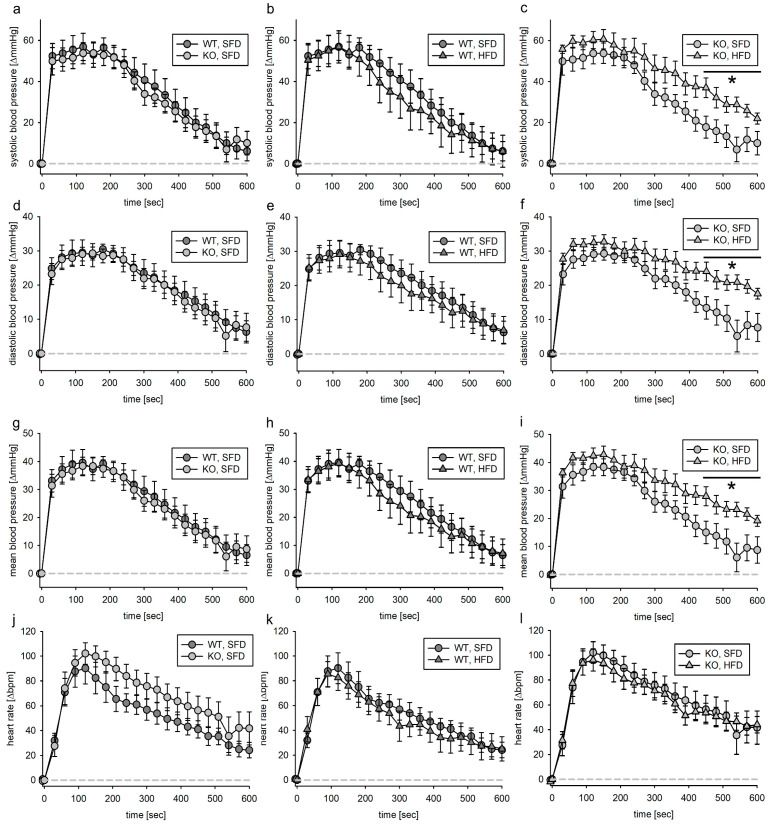
Systolic (**a**–**c**), diastolic (**d**–**f**) and mean (**g**–**i**) blood pressure as well as heart rate (**j**–**l**) response to phenylephrine infusion (1 min) in the EC-EGFR model after subtraction of volume effect. Phenylephrine solution was infused in the jugular vein followed by 1 min vehicle infusion. Blood pressure as well as heart rate were normalized to the respective value within the 5 s before start of infusion. N (WT, SFD) = 11, N (KO, SFD) = 12, N (WT, HFD) = 10, N (KO, HFD) = 9; data given as mean ± SEM; * *p* ≤ 0.05 vs. respective control.

The animals were subdivided into a group treated with PE and a group treated with AII. For both treatments, the dose was given to the animals within one minute. The observation period lasted 20 min. As reported before [14], blood pressure increased upon PE infusion to ~170–190 mmHg systolic and ~100–110 mmHg diastolic. Blood pressure changes were normalized to the blood pressure at the infusion start. As there is a slight impact on volume load in the first minutes of blood pressure increase after substance infusion, we subtracted the mean effect of volume load at each time point of each group from the effect of PE (Figure 4). When comparing the volume-load-corrected PE effect in SFD animals, we found that deletion of the EGFR in ECs had no effect on the blood pressure response. If the EGFR was expressed in ECs, the HFD had no impact on the reactivity to PE. In contrast, as observed for the time point 600–1200 s (Supplementary Figure S3), in KO animals, the blood pressure increase due to PE was more sustained in KO, HFD animals, indicating that the EGFR in endothelial cells is protective against catecholamine (most probably sympathetic nervous system)-induced blood pressure increase in HFD. To analyze if the increase in blood pressure leads to an increased pressure load in the animals, we calculated the vehicle-adjusted AUC for PE. As depicted in Supplementary Figure S4, there was a strong tendency of an increased pressure load for KO, HFD animals compared to their SFD littermates.

#### 3.3.2. SMMHC Animals

In the same way as for the Tie2 animals, the SMMHC animals were subdivided into animals that received PE after vehicle infusion and those that received AII instead. PE increased systolic, diastolic and mean blood pressure as well as heart rate in all animals. In contrast to Tie2 animals, where after vehicle infusion no loss of animals was reported, in SMMHC animals, 20 of 88 animals died upon PE or AII infusion. Of those, 14 animals were KO and 6 were WT. SMMHC animals seem to be more vulnerable to death induced by acute blood pressure increase. As these animals did not reach the end of the observation period, the data were excluded from this analysis.

**Figure 5:**
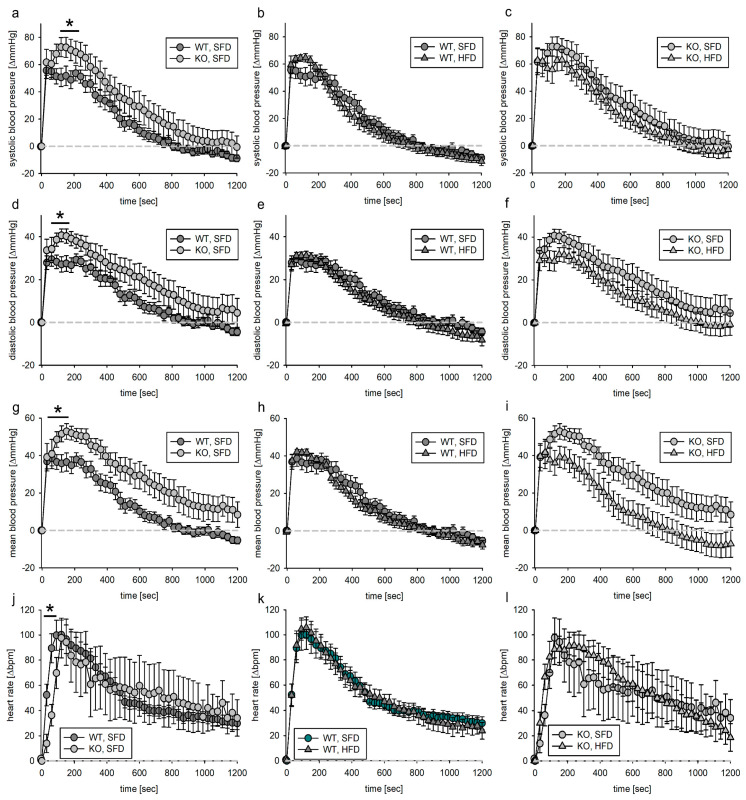
Systolic (**a**–**c**), diastolic (**d**–**f**) and mean (**g**–**i**) blood pressure as well as heart rate (**j**–**l**) response to phenylephrine infusion (1 min) in the VSMC-EGFR model. Phenylephrine solution was infused in the jugular vein followed by 1 min vehicle infusion. Blood pressure as well as heart rate were normalized to the respective value within the 5 s before start of infusion. N(WT, SFD) = 12, N(KO, SFD) = 9, N(WT, HFD) = 9, N(KO, HFD) = 8; data given as mean ± SEM; * *p* ≤ 0.05 vs. respective control.

The maximum blood pressure response of KO, SFD animals to PE infusion was slightly more pronounced regarding systolic, diastolic and mean blood pressure (Figure 5a,d,g) when comparing them to WT, SFD animals. This led to a significantly higher maximum blood pressure increase (Supplementary Figure S5). There was no effect of HFD on blood pressure response to PE infusion, neither in WT nor in KO animals (Figure 5). Neither genotype nor HFD had a major effect on PE-induced heart rate changes (Figure 5j–l).

We calculated the volume-load-adjusted PE effect. The effect of PE within the first 600 s did not differ with respect to genotype or diet (Supplementary Figure S6). However, the increase in heart rate was slightly slower in KO, SFD animals than in WT, SFD animals (Supplementary Figure S6j). This effect was not found in KO animals fed the HFD for 18 weeks (Supplementary Figure S6l). Additionally, the AUC was estimated as a measure of blood pressure load. According to two-way ANOVA, the AUC for mean blood pressure was bigger for KO animals, but this effect was not attributable to a dietary condition (Supplementary Figure S7).

### 3.4. Blood Pressure Response to Acute Angiotensin II Infusion

#### 3.4.1. Tie2 Animals

**Figure 6:**
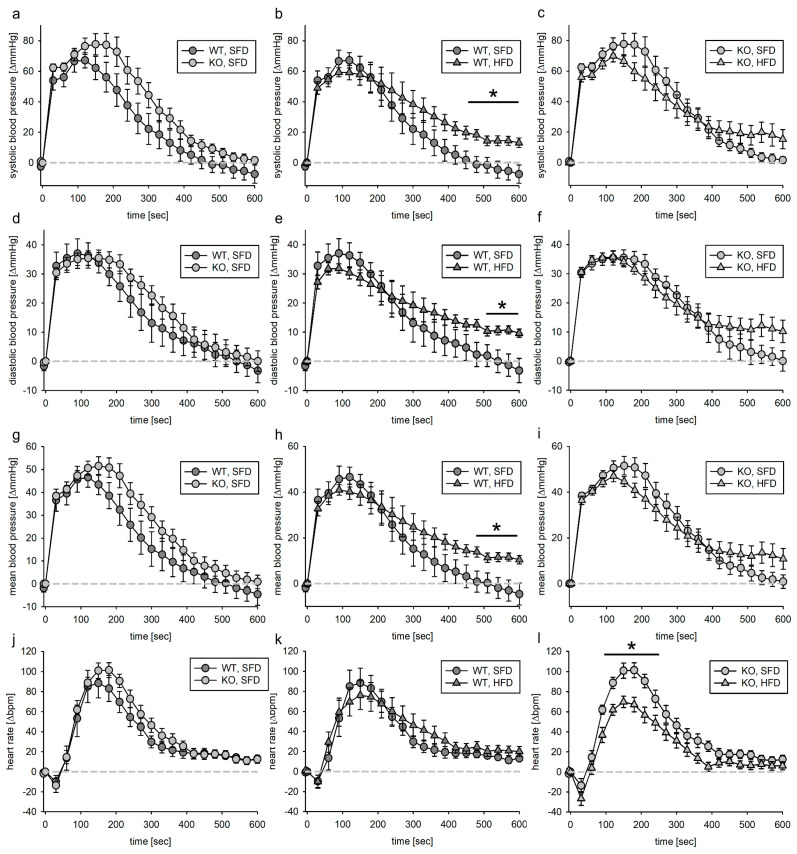
Volume-adjusted systolic (**a**–**c**), diastolic (**d**–**f**) and mean (**g**–**i**) blood pressure as well as heart rate (**j**–**l**) response to angiotensin II infusion (1 min) in the EC-EGFR model. Angiotensin II solution was infused in the jugular vein followed by 1 min vehicle infusion. Blood pressure as well as heart rate were normalized to the respective value within the 5 s before start of infusion, and the mean vehicle effect was subtracted from the respective angiotensin II effect. N(WT, SFD) = 6, N(KO, SFD) = 6, N(WT, HFD) = 7, N(KO, HFD) = 12; data given as mean ± SEM; * *p* ≤ 0.05 vs. respective control.

We infused AII in mice with deletion of the EGFR in ECs. In both genotypes, systolic blood pressure increased about 80 mmHg, diastolic blood pressure increased around 40 mmHg and mean blood pressure increased 50 mmHg (Supplementary Figure S8a,d,g). In both genotypes, the maximum heart rate increase was about ~110 bpm after the start of AII infusion (Supplementary Figure S8j). As there was a slight but significant effect of volume load on blood pressure and heart rate, we compared the effects of AII in the four different groups after subtracting the volume load effect within the first 600 s after the start of AII infusion. There was no significant difference between the two genotypes fed the SFD diet upon AII infusion regarding blood pressure or heart rate (Figure 6a,d,g,j). While the initial peak pressure was the same in wild-type SFD and HFD animals, the blood pressure increase after angiotensin II infusion was prolonged in wild-type HFD animals and to a lesser extent also in KO, HFD animals compared to their SFD littermates (Figure 6b,c,e,f,h,i). The heart rate increase upon AII infusion was reduced in mice lacking the EGFR in endothelial cells and fed with the HFD. The changes in response to AII did not result in a significantly altered blood pressure or heart rate load over time (Supplementary Figure S9).

#### 3.4.2. SMMHC Animals

**Figure 7:**
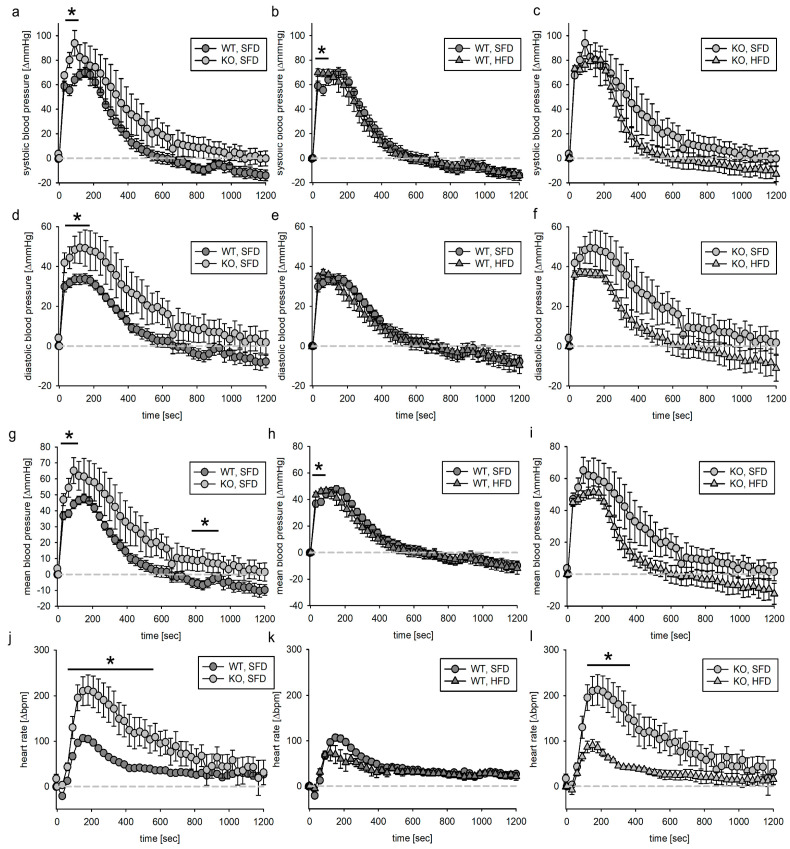
Systolic (**a**–**c**), diastolic (**d**–**f**) and mean (**g**–**i**) blood pressure as well as heart rate (**j**–**l**) response to angiotensin II infusion (1 min) in the VSMC-EGFR model. Angiotensin II solution was infused in the jugular vein followed by 1 min vehicle infusion. Blood pressure as well as heart rate were normalized to the respective value within the 5 s before start of infusion. N(WT, SFD) = 9, N(KO, SFD) = 4, N(WT, HFD) = 13, N(KO, HFD) = 4; data given as mean ± SEM; * *p* ≤ 0.05 vs. respective control.

Blood pressure and heart rate increase upon AII infusion were slightly but significantly higher in KO, SFD than in WT, SFD (Figure 7a,d,g). HFD changed the blood pressure increase in both genotypes only slightly. The heart rate increase was very pronounced in the KO, SFD animals (Figure 7j). This effect was virtually abolished in the KO, HFD animals (Figure 7l).

**Figure 8:**
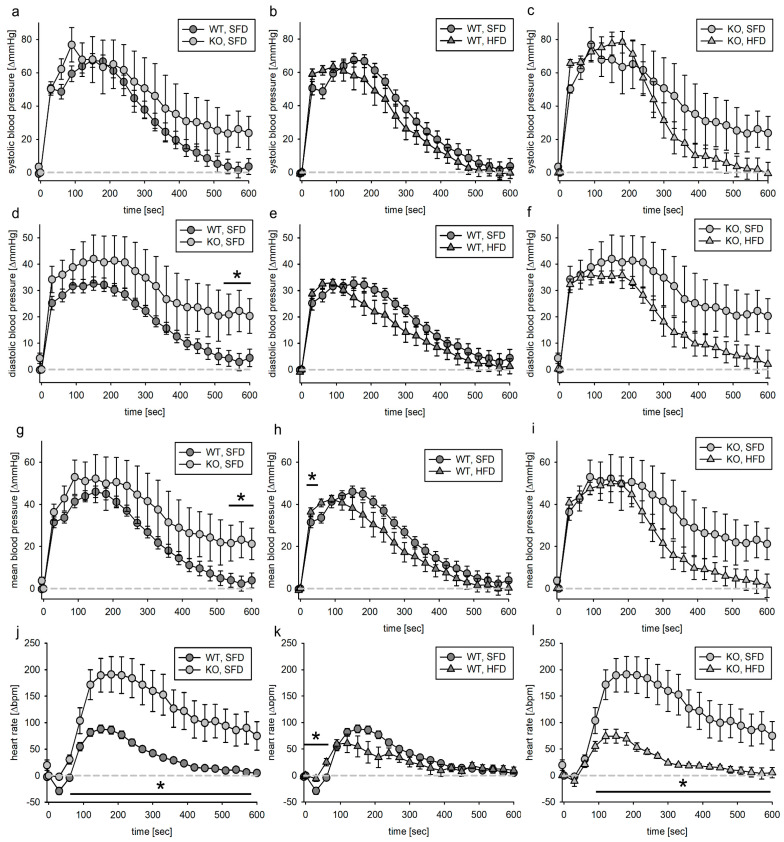
Volume-adjusted systolic (**a**–**c**), diastolic (**d**–**f**) and mean (**g**–**i**) blood pressure as well as heart rate (**j**–**l**) response to angiotensin II infusion (1 min) in the VSMC-EGFR model. Angiotensin II solution was infused in the jugular vein followed by 1 min vehicle infusion. Blood pressure as well as heart rate were normalized to the respective value within the 5 s before start of infusion, and mean vehicle effect was subtracted from the respective angiotensin II effect. N(WT, SFD) = 9, N(KO, SFD) = 4, N(WT, HFD) = 13, N(KO, HFD) = 4; data given as mean ± SEM; * *p* ≤ 0.05 vs. respective control.

To exclude an effect of volume loading on the blood pressure and heart rate increase after AII infusion, we subtracted for the first 600 s the mean blood pressure and heart rate increase from the values obtained after AII infusion. Within the first 600 s of the observation period, there was only a slightly bigger increase in blood pressure of KO, SFD animals compared to WT, SFD animals (Figure 8a,d,g). In SFD animals, heart rate increase upon AII infusion was higher in KO animals than in WT animals (Figure 8j). Compared to SFD, HFD did not alter blood pressure response upon AII stimulation, neither in wild-type nor in KO animals (Figure 8b,c,e,f,h,i). Heart rate increase in KO, HFD animals was in the same range as for the WT animals (Figure 8l). Regarding blood pressure as well as heart rate, the AUC for the KO, SFD animals was bigger than for the wild-type animals and the KO, HFD animals. There was a slight but significant increase in the AUC for the diastolic blood pressure in KO, HFD animals compared to WT, HFD animals (Supplementary Figure S10).

## 4. Discussion

The focus of this article was to describe the impact of the deletion of the EGFR either in VSMCs or in ECs on the acute vasopressor response in animals with or without treatment with a high-fat diet. A major limitation of the study is that the animals were anesthetized. We did so since an acute vascular infusion of vasoconstrictors like phenylephrine or angiotensin II cannot performed without major disturbance of the blood pressure in conscious animals. Therefore, anesthetizing the animals and comparing the effect of the vasopressor to the volume-load-induced blood pressure alteration seems to be the best choice to evaluate the acute, whole-body blood pressure response. To achieve this goal, ketamine/xylazine narcosis was used, producing sufficient anesthesia and pain reduction, but while being cardiovascular depressive [18]. Of course, if one would like to investigate the long-term effect of the two vasopressors on blood pressure and heart rate, telemetry would be the method of choice. Nonetheless, the increase in blood pressure caused by an HFD is comparable to values observed in conscious animals [19].

In the previous section, we reported that a high number of animals in the SMMHC EGFR KO group died during the procedure, and the data needed to be excluded from further analysis. Unfortunately, we did not investigate the cause of death further, but it was recognized that, in some animals, blood could be found in the big body cavities; it needs to be elucidated in further studies whether this finding was a result of altered atherosclerotic lesion formation [20].

### 4.1. Baseline Blood Pressure

Mice with a deletion of the EGFR in endothelial cells develop a slight endothelial dysfunction, which is somewhat stronger in animals fed a high-fat diet [14]. Nonetheless, this slight change is not reflected in the blood pressure homeostasis [14]. HFD induced a slight but significant increase in systolic, diastolic and mean blood pressure regardless of the presence of the EGFR in endothelial cells. 

As reported before [13], postnatal deletion of the EGFR in VSMCs leads to a reduced systolic, diastolic and mean blood pressure, which was confirmed in this study with an additional cohort of animals, thereby demonstrating that the EGFR in VSMCs is important for blood pressure homeostasis under physiological conditions. The HFD caused a slight increase in blood pressure that was more pronounced in mice lacking the EGFR in VSMCs, reaching virtually the same blood pressure values as in mice without deletion of the EGFR in VSMCs. While the EGFR in VSMCs promoted blood pressure increase in a model of chronic RAAS activation [13], it seems to be slightly protective in obesity, as the blood pressure increase is more pronounced if the EGFR is lacking in VSMCs of mice on a high-fat diet. Which factors contribute to the pronounced long-term blood pressure increase in VSMC EGFR KO animals has to be evaluated in more detail. Dicrotic notch pressure increased in KO animals but not in WT animals, indicating a hypertension component of resistance, fit with the previously reported results. While the slightly reduced baseline blood pressure increase is in contrast to our findings from a previous publication [16], the increase in dicrotic notch pressure supports the previous findings. It has to be investigated whether the divergent results are due to, e.g., compensatory mechanisms. 

Additionally, lack of EGFR in either one of the two cell types leads to an increase in heart rate upon an HFD that is stronger than in the corresponding WT animals. It needs to be investigated in further studies whether the deletion of the EGFR in either VSMCs or ECs influences the heart function under a high-fat diet. The increase in heart rate might be a consequence of a slightly higher sympathoexcitatory effect induced by obesity and metabolic disorder [1]. To evaluate the possible underlying mechanism was beyond the scope of this study.

Previous studies have reported increased activity of the sympathetic nervous system as well as the renin–angiotensin–aldosterone-system in obese animals as well as in obese humans [1,21,22,23,24]. Several studies have investigated ex vivo the vascular reactivity of vessels from obese animals stemming from various locations. Their results are somewhat conflicting. For example, while an overall increase in vasopressive agents has been reported in mesenteric arteries of diet-induced hypertensive rats [19], a reduced vasoconstrictor response upon phenylephrine stimulation of aortic rings in rats fed a high-fat and high-sucrose diet was observed [1]. Blood pressure is under constant fluctuation, leading to regulatory and counter-regulatory actions that need to be balanced. Balancing includes not only different organs like the heart, blood vessels and the nervous system but also a huge variety of vasoactive substances, like, e.g., epoxyeicosanoids, prostanoids, potassium channels modulators and endothelin. Therefore, we believe that an integrative view, analyzing the virtually intact cardiovascular system with its control organs, is needed.

### 4.2. Vehicle Loading

In the EC model, the blood pressure in the WT, SFD, WT, HFD and KO, SFD groups returns to starting values 150–200 s after volume infusion, while the blood pressure in the KO, HFD animals drops to values below the starting volumes. This indicates a disturbed counter-regulatory mechanism, overachieving blood pressure reduction or a reduced stabilizing mechanism. Most probably, neither the baroreceptor reflex nor the long-term blood-pressure-regulating mechanisms are causative, as the time frame for neither is fitting [25,26,27,28]. We cannot exclude that deletion of the EGFR in ECs might lead to differences in the reactivity of the venous system, which would counter an immediate increase in blood pressure through increased relaxation [27]. In summary, the EGFR in ECs is of minor importance for volume-load-induced blood pressure changes.

In mice with a deletion of the EGFR in VSMCs, volume load induced a significantly stronger increase in systolic, diastolic and mean blood pressure compared to their wild-type littermates. In a previous study, we analyzed the blood pressure response eight weeks after deletion of the EGFR in VSMCs [13]. At this time point, a tissue-dependent heterogeneity in the vessel morphology with a slightly dilated vascular phenotype in some but not all tissues that change with age was observed. Deletion of the EGFR in VSMCs causes a shift from the contractile phenotype to a more migratory/secretory phenotype producing more extracellular matrix [29]. The prolonged deletion of the EGFR in this study might have led to an enhanced accumulation of fibrotic tissue, which could have induced an increase in blood pressure response upon volume load. As in the EC EGFR WT animals, HFD did not result in an altered blood pressure response upon volume load and blood pressure.

### 4.3. Phenylephrine

Infusion of phenylephrine leads to a substantial and prolonged increase in blood pressure that is not different between animals with and without deletion of the EGFR in ECs fed the SFD. This is astonishing as a slight but significant sensitization of mesenteric arteries to phenylephrine in pressure myography was observed [14]. This increase in sensitivity corresponds to a shift in the concentration, leading to a half-maximal vasoconstriction from 1 µM to 0.3 µM in EC EGFR KO mice. In the in vivo experiments, we infused 100 µg PE/kg BW. This would correspond to a molar concentration of ~0.5 µmol PE, which would be slightly higher than the reported EC50 for aortic rings from C57Bl6 mice [30] and would relate to the previously reported half-maximal vasoconstriction induced in aortic rings of the EC EGFR strain [14]. Therefore, one would expect at least a slight difference in blood pressure response. We conclude that although there are at least regional alterations in the vascular reactivity, compensatory mechanisms ensure that short-term global blood pressure regulation upon adrenergic stimulation does not depend on the EGFR in endothelial cells in lean animals.

In wild-type animals, an HFD does not cause an alteration in the vascular response to PE. This is in contrast with the literature [19,31,32,33,34,35]. Although there is at least one report demonstrating that while there is an exaggerated pressor response upon norepinephrine in obese Zucker rats, this is not true for phenylephrine [36]. There are studies explaining that changes in isolated vascular reactivity seem to be compensated for by the baroreceptor reflex or other mechanisms [35]. While the blood pressure returns to baseline volumes after 10 min for the WT and KO, SFD animals, the blood pressure is still increased in EGFR EC KO mice fed the HFD. The persistent increase in blood pressure could be a result of a reduced vasodilator response seen in vitro [14], especially as the effect seems to be more pronounced on diastolic and mean blood pressure, both reflecting the resistance/vessel component of the arterial blood pressure. In summary, deletion of the EGFR in ECs leads to slightly prolonged blood pressure increase upon adrenergic stimulation in DMT2 animals.

In mice with deletion of the EGFR in VSMCs, blood pressure increase within the first 200 s was higher compared to WT animals under the SFD. The HFD did not cause an alteration in blood pressure response in WT or in KO animals. When comparing the area under the curve, as a measure for the overall blood pressure load, the difference in blood pressure load is attributable to the lack of EGFR, not to the diet. Therefore, the EGFR in VSMCs seems to modulate vascular reactivity upon adrenergic stimulation regardless of the diet [19,31,32,33,34,35].

### 4.4. Angiotensin II

Deletion of the EGFR in ECs did not alter the blood pressure response upon AII infusion compared to WT animals receiving an SFD chow. The HFD induced a slightly but significantly prolonged blood pressure increase upon AII infusion in mice without deletion but not in mice with deletion of the EGFR in ECs. The prolonged blood pressure increase could be the result of reduced vasodilation, as was reported before [7,14]. In this case, the EGFR in ECs would promote vasorelaxation in HFD-fed animals but not SFD-fed animals. Belmadani et al. [7] demonstrated, in mesenteric resistance arteries from diabetic mice (db/db strain), that total and phosphorylated eNOS protein content was normalized to control levels if the animals were treated with AG1478, an EGFR inhibitor. Unfortunately, a group demonstrating the effect of AG1478 alone was not included in this study. In summary, EC EGFR is not important for vascular reactivity upon angiotensin II stimulation in lean animals but it might exhibit a slight protective effect in obese animals.

The effect of the blood pressure response to acute AII exposure in VSMC-EGFR KO animals is in the first place surprising. There have been numerous reports that the deletion/inhibition of the EGFR attenuates the reaction of blood vessels to AII [13,15,24,37,38,39]. In this model, we show that the lack of the EGFR in VSMCs in lean mice leads to a stronger response to AII infusion. However, one has to take into account the different changes in heart rate, which is the main component of acute changes in cardiac output. The relation of the relative changes in blood pressure and the changes in heart rate, i.e., cardiac output (ΔTPR~ΔBP/ΔHR), indicates an increase in total peripheral resistance (TPR) for the WT animals (ΔBP/ΔHR~1.44), while it indicates only a minor change in TPR in KO animals (ΔBP/ΔHR~0.89). This confirms our previous observation that force increase upon angiotensin stimulation is reduced in mice lacking the EGFR in VSMCs [13]. As a consequence, the more pronounced increase in heart rate is a compensatory mechanism to maintain the blood pressure response.

The HFD had only minor effects on the acute blood pressure response upon angiotensin stimulation in the VSMC-EGFR WT mice; this is somewhat surprising as there was a slight but significantly prolonged increase in blood pressure observed in the EC-EGFR strain. As mentioned above, those two strains differ slightly regarding their genetic background, leading to a “profibrotic” transcriptomic change in EC-EGFR WT compared to VSMC EGFR WT animals [14]. This transcriptomic change might lead to enhanced vascular fibrosis in the EC-EGFR WT animals that is intensified by an HFD. In the VSMC EGFR KO mice fed an HFD, the blood pressure response and heart rate response were comparable to the wild-type animals. These findings are in good agreement with transcriptomic data from these animals, where the HFD-induced changes that were observed in the WT animals were virtually abolished in the KO animals [16]. This does not exclude that there is an effect of the VSMC EGFR in an HFD, as a major impact of angiotensin II in obesity is on the perfusion of the microvasculature [40] and these vessels were not the scope of our study. 

## 5. Conclusions

DMT2 and/or MetS are frequently associated with hypertension. In recent years, it has been demonstrated that the vascular EGFR is involved in the cardiovascular changes elicited by DMT2. In this study, we demonstrated that VSMCs are the main cell type responsible for the short-term vascular impact of the EGFR. We confirmed in an independent cohort that deletion of the VSMC-EGFR leads to a reduced blood pressure, followed by an increased heart rate to compensate for the reduced blood pressure. Regarding vasoactive substances, the EC-EGFR seems to have no importance for angiotensin II action and counteracting HFD-induced prolonged blood pressure increase upon PE stimulation. The VSMC-EGFR supports the blood pressure response to adrenergic and angiotensin II stimulation in lean animals. The responsiveness to AII and alpha-adrenergic stimulation was similar in lean and obese animals despite the known enhanced activity of the RAAS and the sympathetic nervous system under a high-fat diet. This could represent a situation of a relative enhanced responsiveness. The underlying mechanism needs to be analyzed, as several vasoactive factors like epoxyeicosatrienoids, prostanoids and potassium channel expression, beside impaired nitric oxide production, are altered by a high-fat diet in mice.

## Data Availability

Not applicable.

## Supplementary Materials

The following supporting information can be downloaded at: https://www.mdpi.com/article/10.3390/biomedicines11082241/s1, Figure S1: Body weight (a,b) and dicrotic notch pressure (c,d) in mice with (KO) or without (WT) deletion of the EGFR in endothelial cells (EC, (a,c)) or vascular smooth muscle cellt (VSMC, b, d) after 18 weeks fed with standard fat diet (SFD) or high fat diet (HFD). Body weight: EC-model N(WT, SFD) = 10, N(KO, SFD) = 9, N(WT, HFD) = 8, N(KO, HFD) = 12; VSMC-model N(WT, SFD) = 30, N(KO, SFD) = 32, N(WT, HFD) = 28, N(KO, HFD) = 38, Dicrotic notch pressure EC-model N(WT, SFD) = 10, N(KO, SFD) = 9, N(WT, HFD) = 8, N(KO, HFD) = 12; VSMC-model N(WT, SFD) = 18, N(KO, SFD) = 22, N(WT, HFD) = 21, N(KO, HFD) = 27, * *p* ≤ 0.05 vs. respective control.; Figure S2: Area under the curve (AUC) of the blood pressure change induced by volume load in mice with (KO) or without (WT) deletion of the EGFR in VSMC. AUC was calculated with the trapezoid formula and measured until blood pressure or heart rate reached the initial volumes. N(WT, SFD) = 25, N(KO, SFD) = 15, N(WT, HFD) = 24, N(KO, HFD) = 17; Data given as mean ± SEM; * *p* ≤ 0.05 vs. respective control; Figure S3: Systolic (a–c), diastolic (d–f) and mean (g–i) blood pressure as well as heart rate (j–l) response on phenylephrine infusion (1 min) in the EC-EGFR-model. Phenylephrine solution was infused in the jugular vein followed by 1 min vehicle infusion. Blood pressure as well as heart rate was normalized to the respective value within the 5 s before start of infusion. N (WT, SFD) = 11, N (KO, SFD) = 12, N (WT, HFD) = 10, N (KO, HFD) = 9; Data given as mean ± SEM; * *p* ≤ 0.05 vs. respective control; Figure S4: Area under the curve (AUC) of the vehicle-adjusted blood pressure change induced by phenylephrine in mice with (KO) or without (WT) deletion of the EGFR in EC. AUC was calculated with the trapezoid formula and measured until blood pressure or heart rate reached the initial volumes or maximum within 600 s after substance application. N(WT, SFD) = 10, N(KO, SFD) = 10, N(WT, HFD) = 24, N(KO, HFD) = 9; Data given as mean ± SEM; Figure S5: Maximum blood pressure and heart rate increase upon PE infusion in mice with (KO) or without (WT) deletion of the EGFR in VSMC. N(WT, SFD) = 12, N(KO, SFD) = 9, N(WT, HFD) = 9, N(KO, HFD) = 8; Data given as mean ± SEM; * *p* ≤ 0.05 vs. respective control; Figure S6: Volume-adjusted systolic (a–c), diastolic (d–f) and mean (g–i) blood pressure as well as heart rate (j–l) response on phenylephrine infusion (1min) in the VSMC-EGFR-model after subtraction of volume effect. Phenylephrine solution was infused in the jugular vein followed by 1 min vehicle infusion. Blood pressure as well as heart rate was normalized to the respective value within the 5 s before start of infusion. N(WT, SFD) = 12, N(KO, SFD) = 9, N(WT, HFD) = 8, N(KO, HFD) = 8; Data given as mean ± SEM; * *p* ≤ 0.05 vs. respective control; Figure S7: Area under the curve (AUC) of the vehicle-adjusted blood pressure change induced by phenylephrine in mice with (KO) or without (WT) deletion of the EGFR in VSMC. AUC was calculated with the trapezoid formula and measured until blood pressure or heart rate reached the initial volumes or maximum within 600 s after substance application. N(WT, SFD) = 12, N(KO, SFD) = 9, N(WT, HFD) = 9, N(KO, HFD) = 8; Data given as mean ± SEM; Figure S8: Systolic (a–c), diastolic (d–f) and mean (g–i) blood pressure as well as heart rate (j–l) response on angiotensin II infusion (1min) in the EC-EGFR-model. Angiotensin II solution was infused in the jugular vein followed by 1 min vehicle infusion. Blood pressure as well as heart rate was normalized to the respective value within the 5 s before start of infusion. N(WT, SFD) = 6, N(KO, SFD) = 6, N(WT, HFD) = 7, N(KO, HFD) = 12; Data given as mean ± SEM; * *p* ≤ 0.05 vs. respective control. Figure S9: Area under the curve (AUC) of the vehicle-adjusted blood pressure change induced by angiotensin II in mice with (KO) or without (WT) deletion of the EGFR in EC. AUC was calculated with the trapezoid formula and measured until blood pressure or heart rate reached the initial volumes or maximum within 600 s after substance application. N(WT, SFD) = 6, N(KO, SFD) = 6, N(WT, HFD) = 7, N(KO, HFD) = 12; Data given as mean ± SEM; Figure S10: Area under the curve (AUC) of the vehicle-adjusted blood pressure change induced by angiotensin II in mice with (KO) or without (WT) deletion of the EGFR in VSMC. AUC was calculated with the trapezoid formula and measured until blood pressure or heart rate reached the initial volumes or maximum within 600 s after substance application. N(WT, SFD) = 9, N(KO, SFD) = 4, N(WT, HFD) = 13, N(KO, HFD) = 4; Data given as mean ± SEM; * *p* ≤ 0.05 vs. respective control.
