# Supplementary material for: Assessment of the Role of Endothelial and Vascular Smooth Muscle EGFR for Acute Blood Pressure Effects of Angiotensin II and Adrenergic Stimulation in Obese Mice
Source: Biomedicines. 2023 Aug 9;11(8):2241. doi: 10.3390/biomedicines11082241 (PMC10452314; doi:10.3390/biomedicines11082241)
Supplement: Supplementary file 1 [file biomedicines-11-02241-s001.zip › biomedicines-2537351-supplementary.pdf]

Supplementary Materials

Supplementary Figure S1

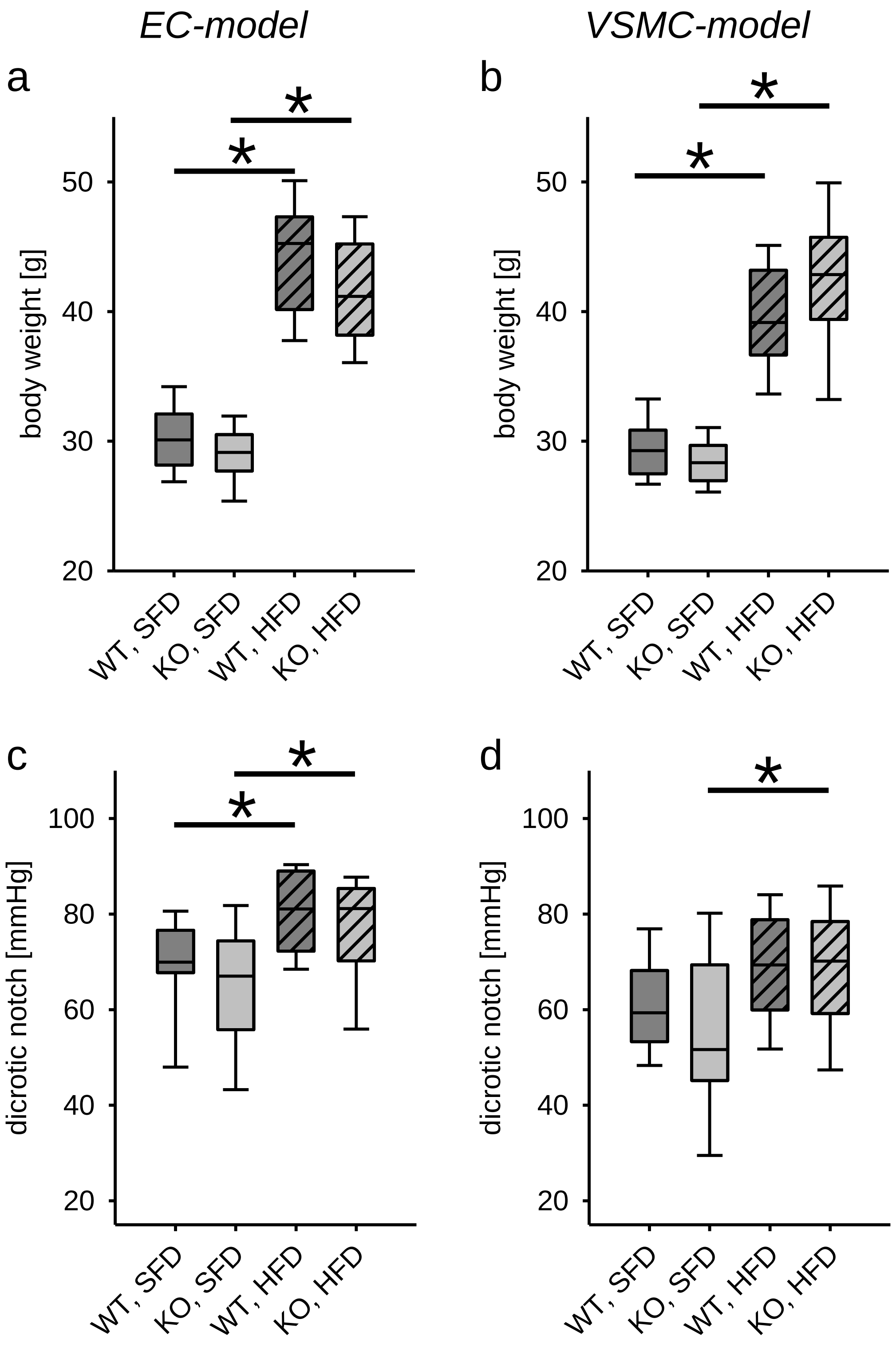

Figure S1. Body weight (**a**,**b**) and dicrotic notch pressure (**c**,**d**) in mice with (KO) or without (WT) deletion of the EGFR in endothelial cells (EC (**a**, **c**)) or vascular smooth muscle cellt (VSMC, b, d) after 18 weeks fed with standard fat diet (SFD) or high fat diet (HFD). Body weight: EC-model N(WT, SFD)= 10, N(KO, SFD)=9, N(WT, HFD)=8, N(KO, HFD)=12; VSMC-model N(WT, SFD)= 30, N(KO, SFD)=32, N(WT, HFD)=28, N(KO, HFD)=38, Dicrotic notch pressure EC-model N(WT, SFD)= 10, N(KO, SFD)=9, N(WT, HFD)=8, N(KO, HFD)=12; VSMC-model N(WT, SFD)= 18, N(KO, SFD)=22, N(WT, HFD)=21, N(KO, HFD)=27, * p≤0.05 vs. respective control.

Supplementary Figure S2

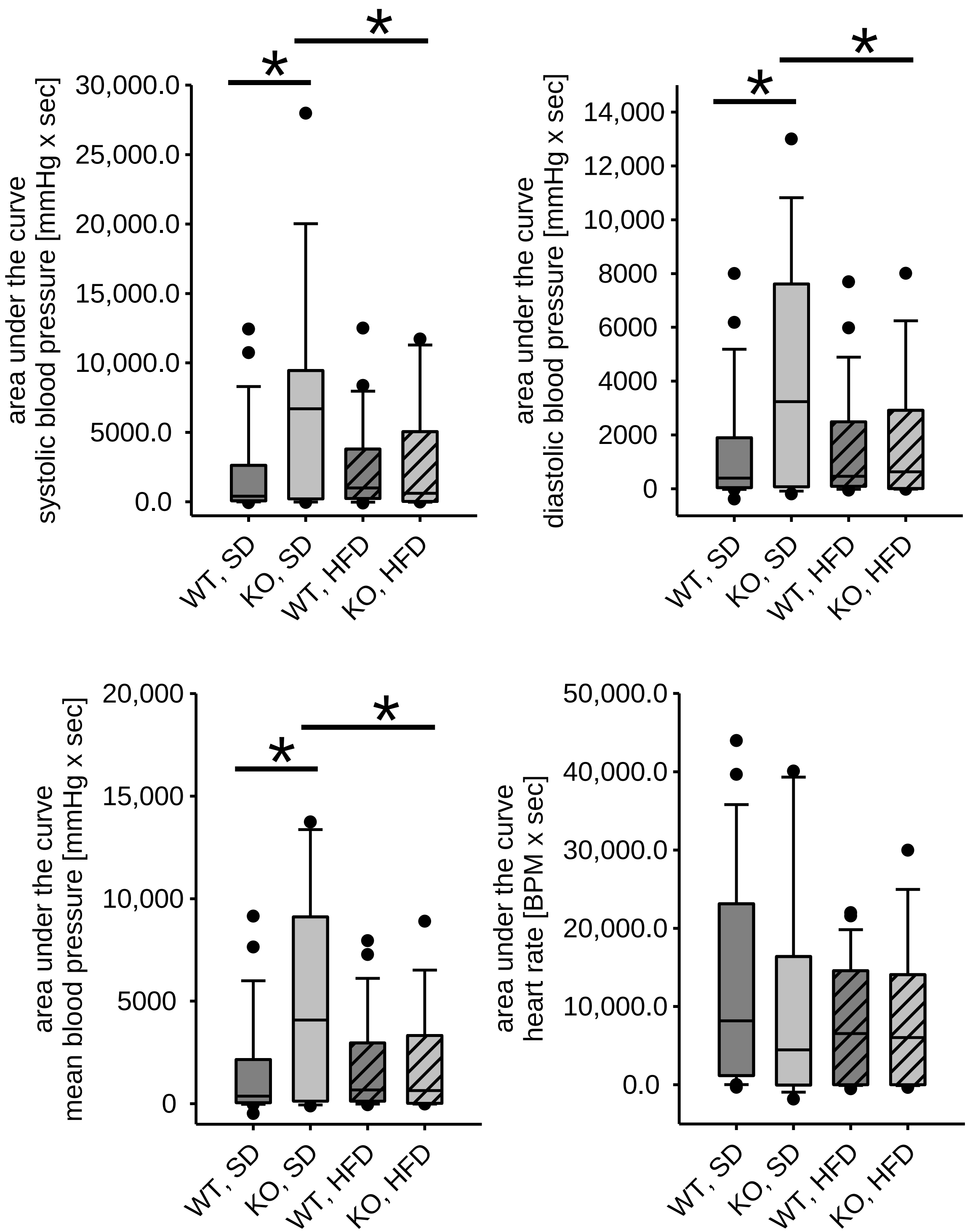

Figure S2. Area under the curve (AUC) of the blood pressure change induced by volume load in mice with (KO) or without (WT) deletion of the EGFR in VSMC. AUC was calculated with the trapezoid formula and measured until blood pressure or heart rate reached the initial volumes. N(WT, SFD)= 25, N(KO, SFD)=15, N(WT, HFD)=24, N(KO, HFD)=17; Data given as mean ± SEM; * p≤0.05 vs. respective control .

Supplementary Figure S3 (Part I)

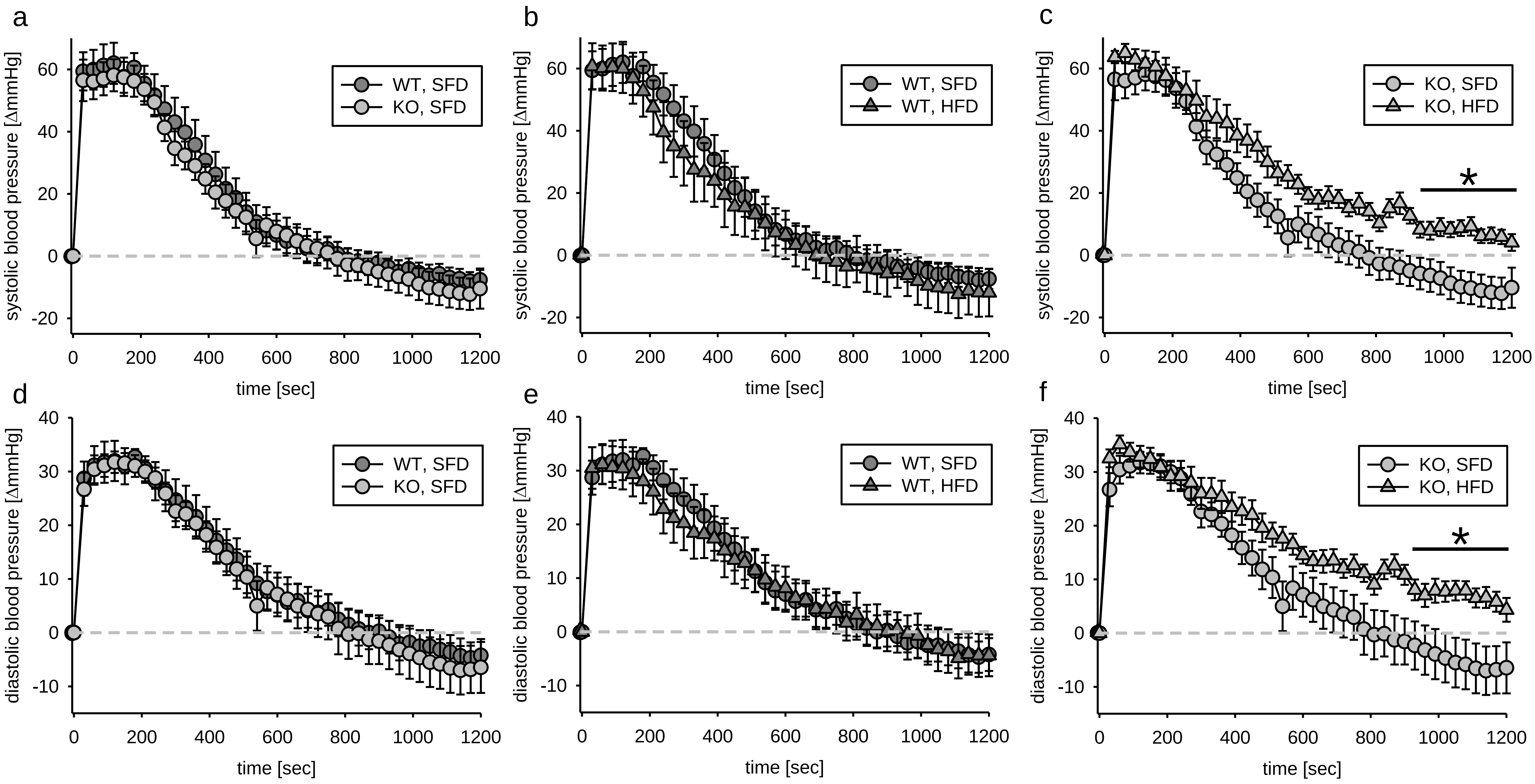

Supplementary Figure S3 (Part II)

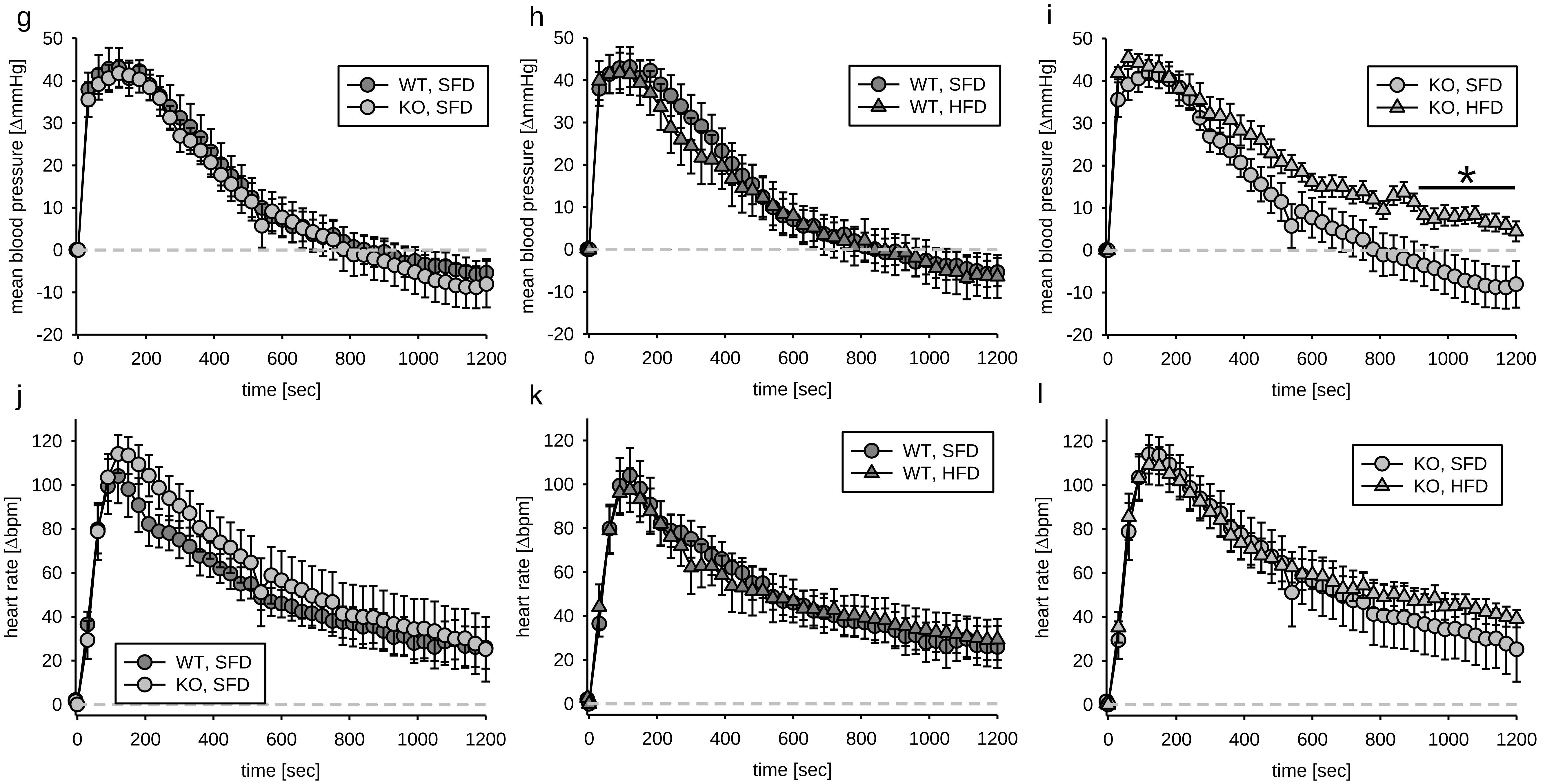

Figure S3. Systolic (**a-c**), diastolic (**d-f**) and mean (**g-i**) blood pressure as well as heart rate (**j-l**) response on phenylephrine infusion (1 min) in the EC-EGFR-model. Phenylephrine solution was infused in the jugular vein followed by 1 min vehicle infusion. Blood pressure as well as heart rate was normal-ized to the respective value within the 5 seconds before start of infusion. N (WT, SFD) = 11, N (KO, SFD) = 12, N (WT, HFD) = 10, N (KO, HFD) = 9; Data given as mean ± SEM; * p ≤ 0.05 vs. respective control.

Supplementary Figure S4

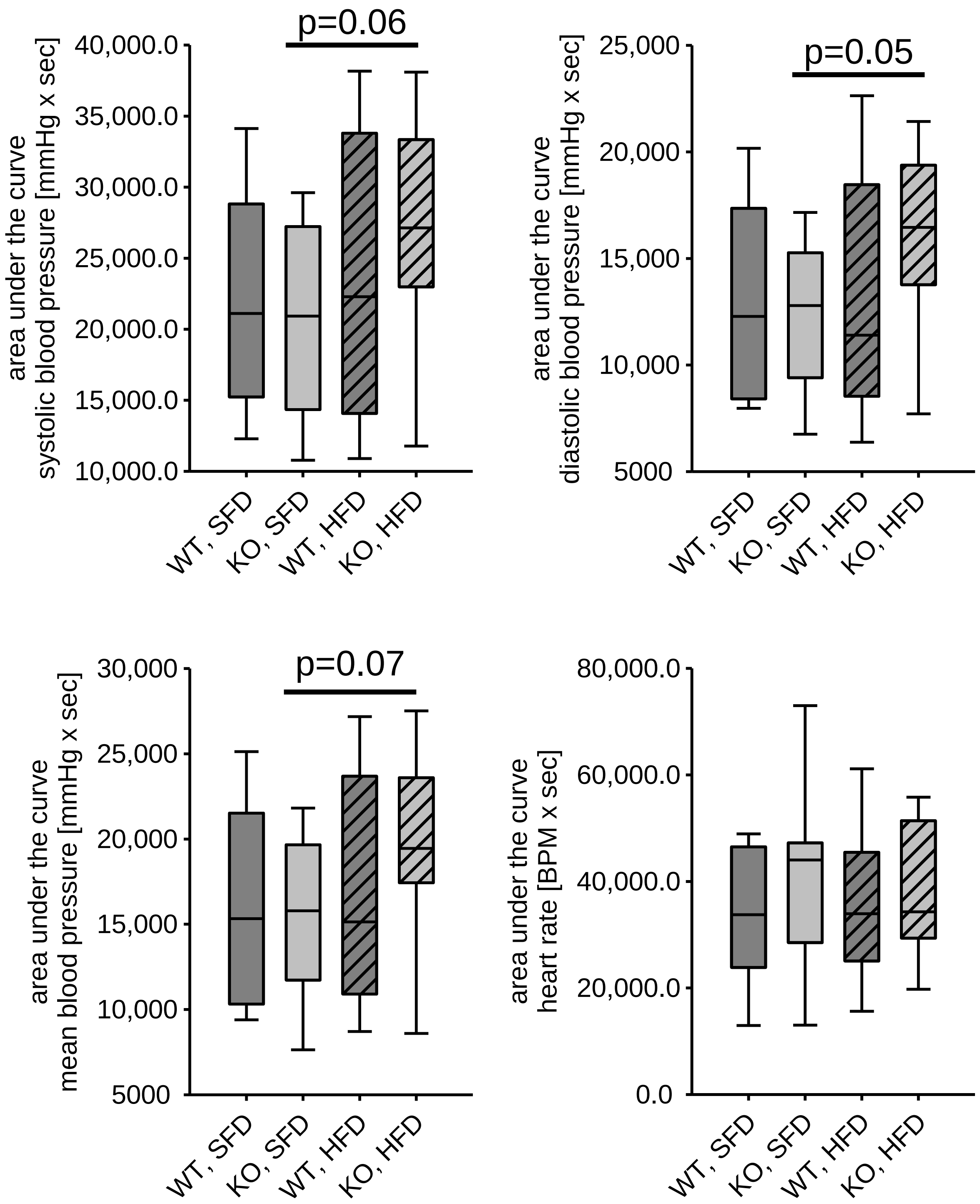

Figure S4. Area under the curve (AUC) of the vehicle-adjusted blood pressure change induced by phenylephrine in mice with (KO) or without (WT) deletion of the EGFR in EC. AUC was calculated with the trapezoid formula and measured until blood pressure or heart rate reached the initial volumes or maximum within 600 seconds after substance application. N(WT, SFD)= 10, N(KO, SFD)=10, N(WT, HFD)=24, N(KO, HFD)=9; Data given as mean ± SEM.

Supplementary Figure S5

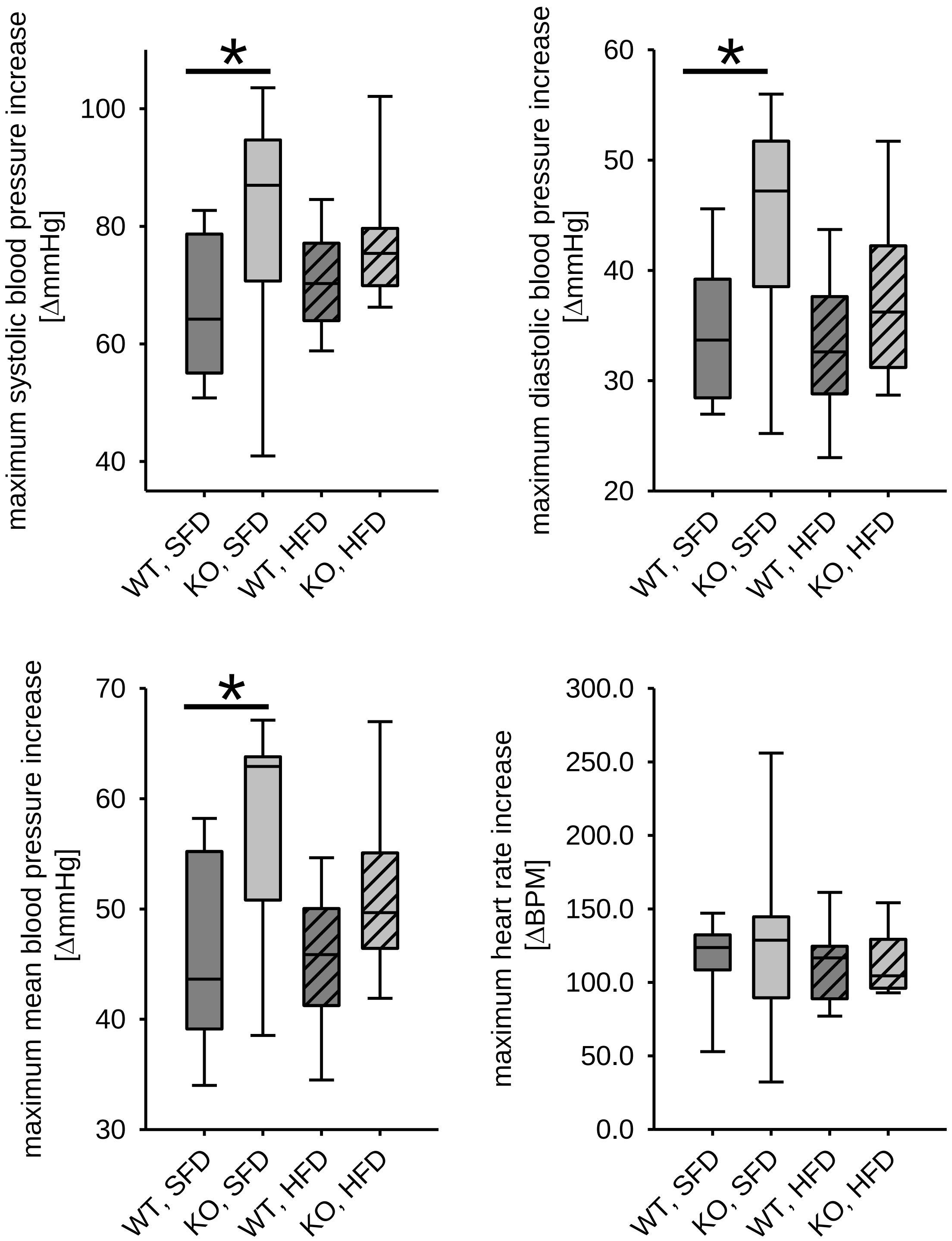

Figure S5. Maximum blood pressure and heart rate increase upon PE infusion in mice with (KO) or without (WT) deletion of the EGFR in VSMC. N(WT, SFD)= 12, N(KO, SFD)=9, N(WT, HFD)=9, N(KO, HFD)=8; Data given as mean ± SEM; * p ≤ 0.05 vs. respective control.

Supplementary Figure S6 (Part I)

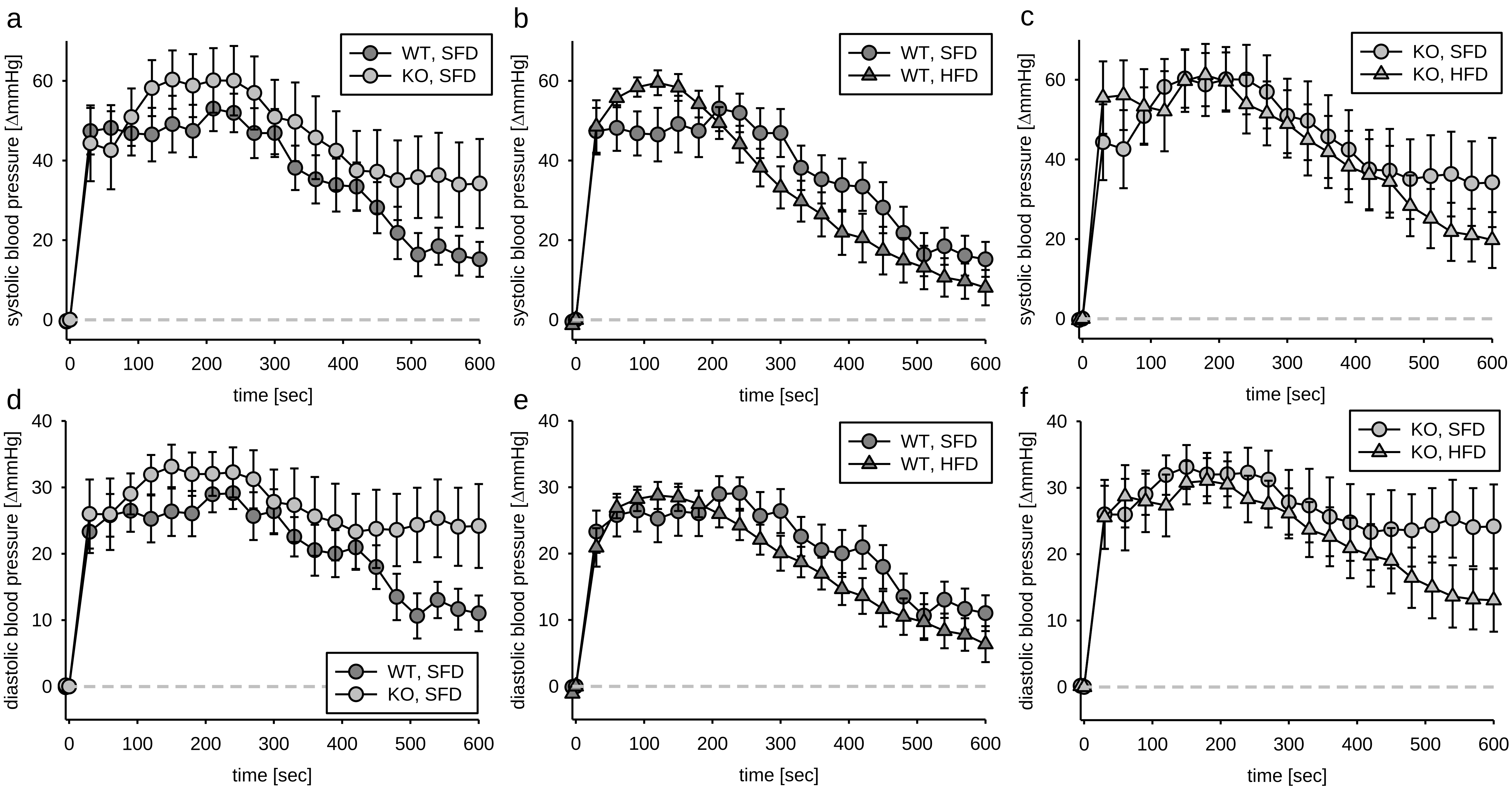

Supplementary Figure S6 (Part II)

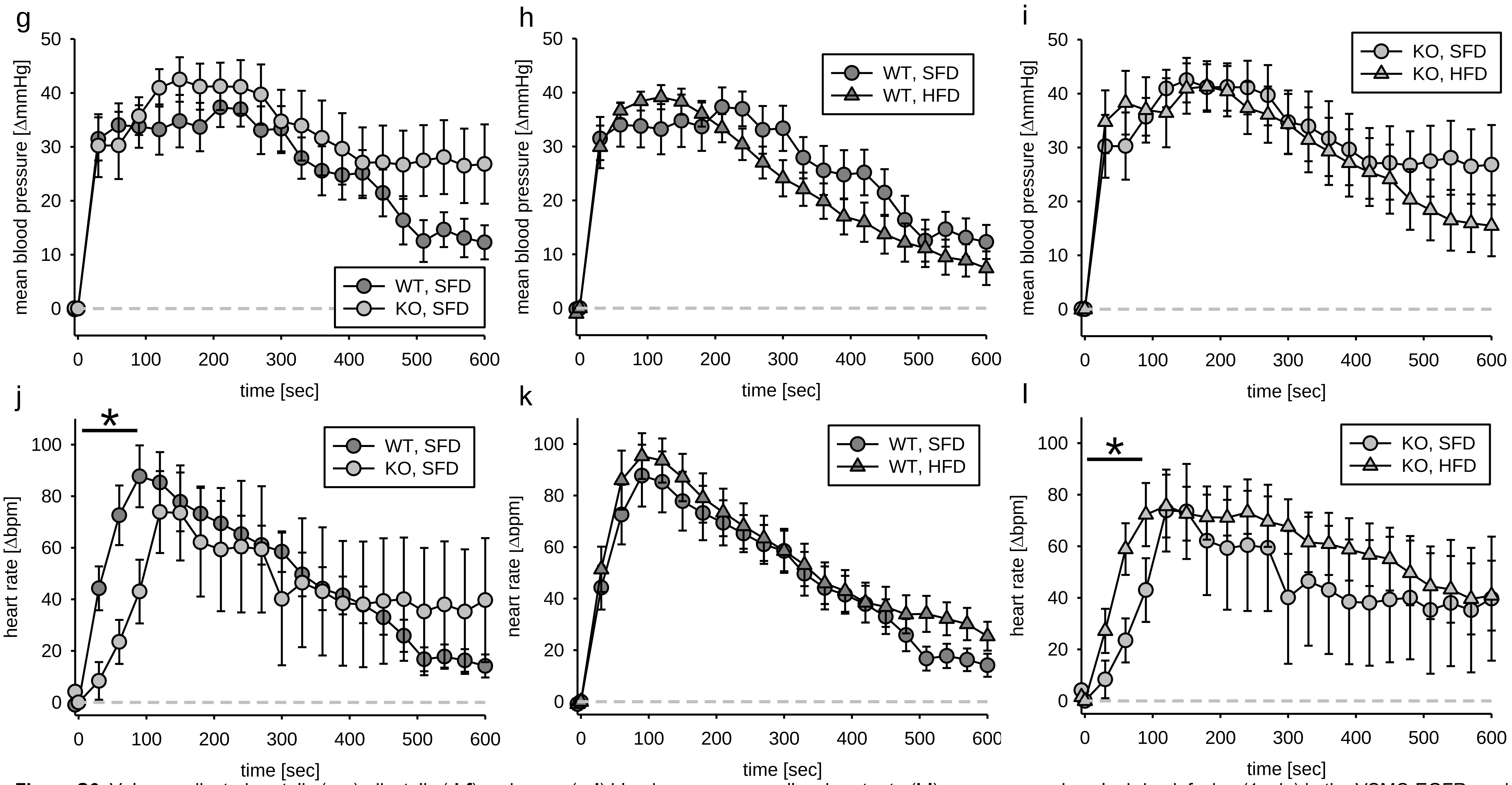

Figure S6. Volume-adjusted systolic (**a-c**), diastolic (**d-f**) and mean (**g-i**) blood pressure as well as heart rate (**j-l**) response on phenylephrine infusion (1 min) in the VSMC-EGFR-model. Phenylephrine solution was infused in the jugular vein followed by 1 min vehicle infusion. Blood pressure as well as heart rate was normalized to the respective value within the 5 seconds before start of infusion. N(WT, SFD)= 12, N(KO, SFD)=9, N(WT, HFD)=8, N(KO, HFD)=8; Data given as mean ± SEM; * p ≤ 0.05 vs. respective control.

Supplementary Figure S7

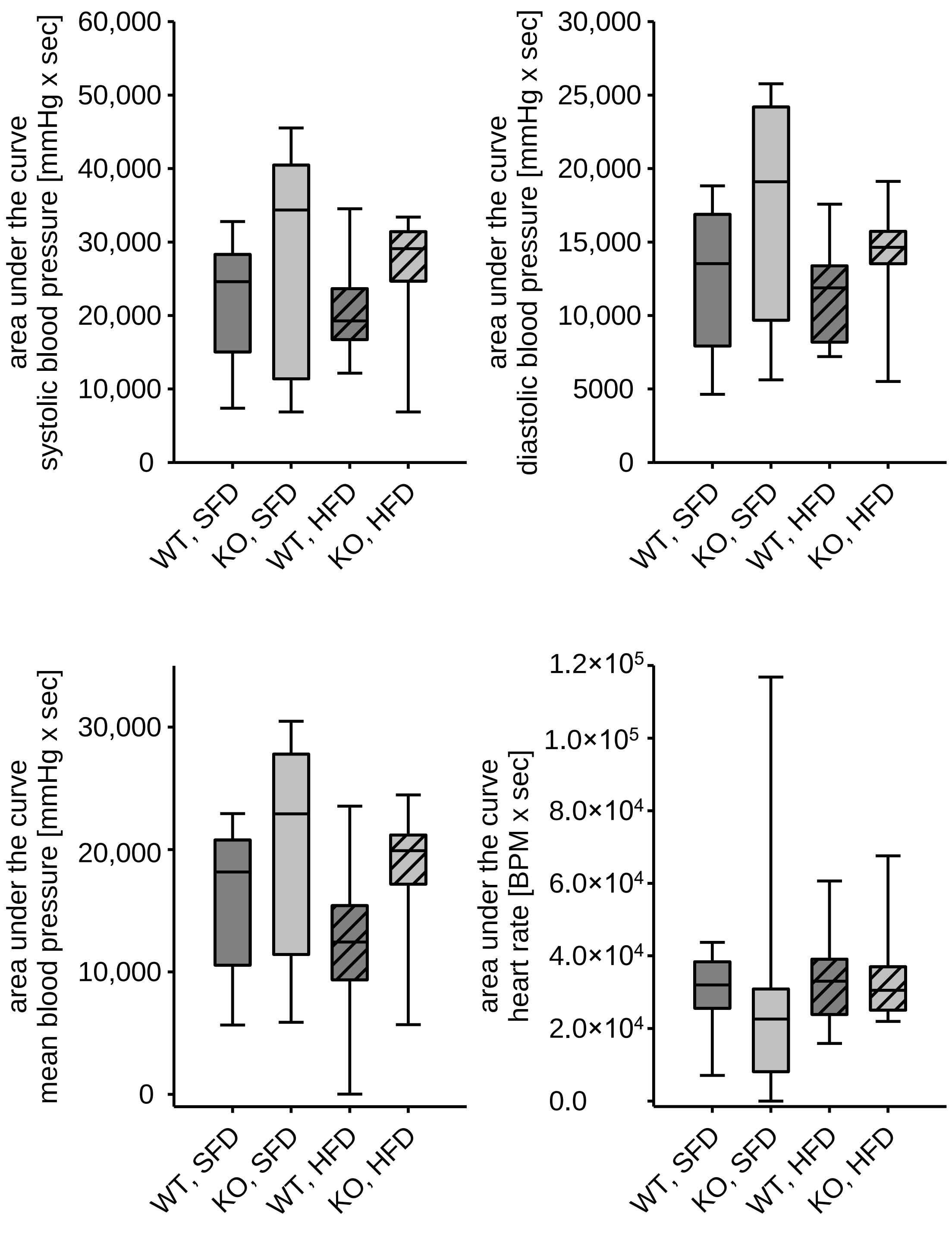

Figure S7. Area under the curve (AUC) of the vehicle-adjusted blood pressure change induced by phenylephrine in mice with (KO) or without (WT) deletion of the EGFR in VSMC. AUC was calculated with the trapezoid formula and measured until blood pressure or heart rate reached the initial volumes or maximum within 600 seconds after substance application. N(WT, SFD)= 12, N(KO, SFD)=9, N(WT, HFD)=9, N(KO, HFD)=8; Data given as mean ± SEM.

Supplementary Figure S8 (Part I)

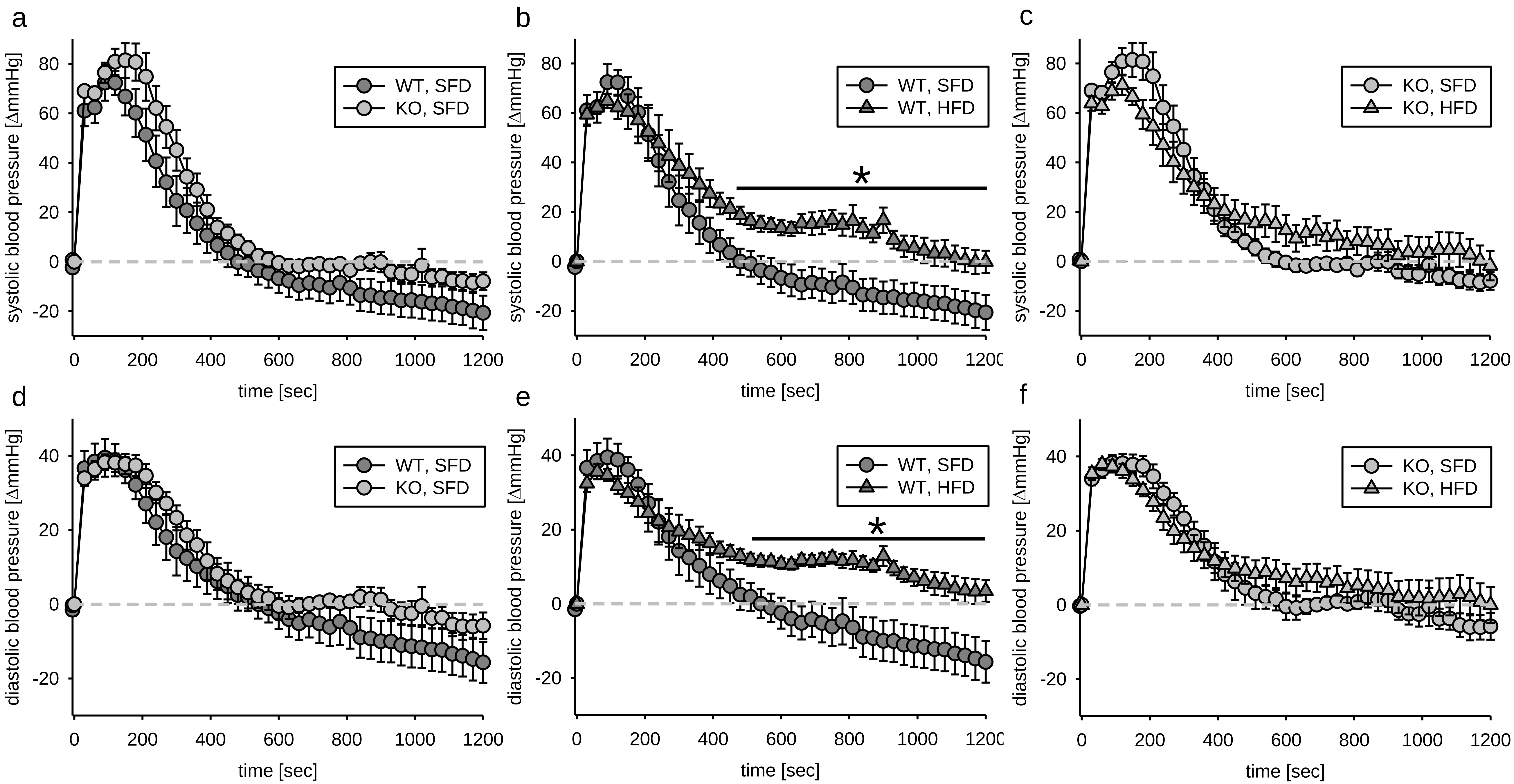

Supplementary Figure S8 (Part II)

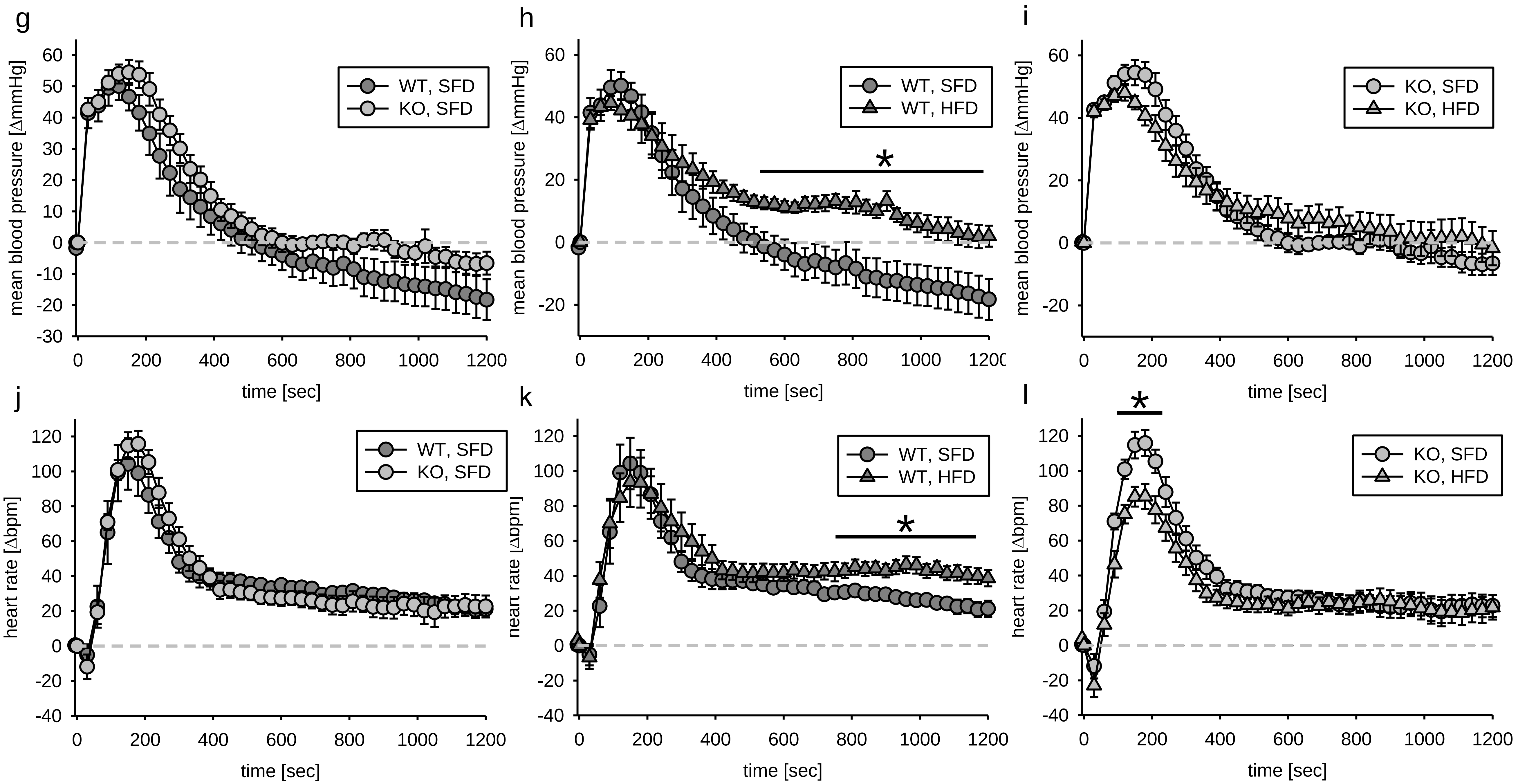

Figure S8. Systolic (**a**-**c**), diastolic (**d**-**f**) and mean (**g-i)** blood pressure as well as heart rate (**j**-**l**) response on angiotensin II infusion (1min) in the EC-EGFR-model. Angiotensin II solution was infused in the jugular vein followed by 1 min vehicle infusion. Blood pressure as well as heart rate was normalized to the re-spective value within the 5 seconds before start of infusion. N(WT, SFD)= 6, N(KO, SFD)=6, N(WT, HFD)=7, N(KO, HFD)= 12; Data given as mean ± SEM; * p ≤ 0.05 vs. respective control.

Supplementary Figure S9

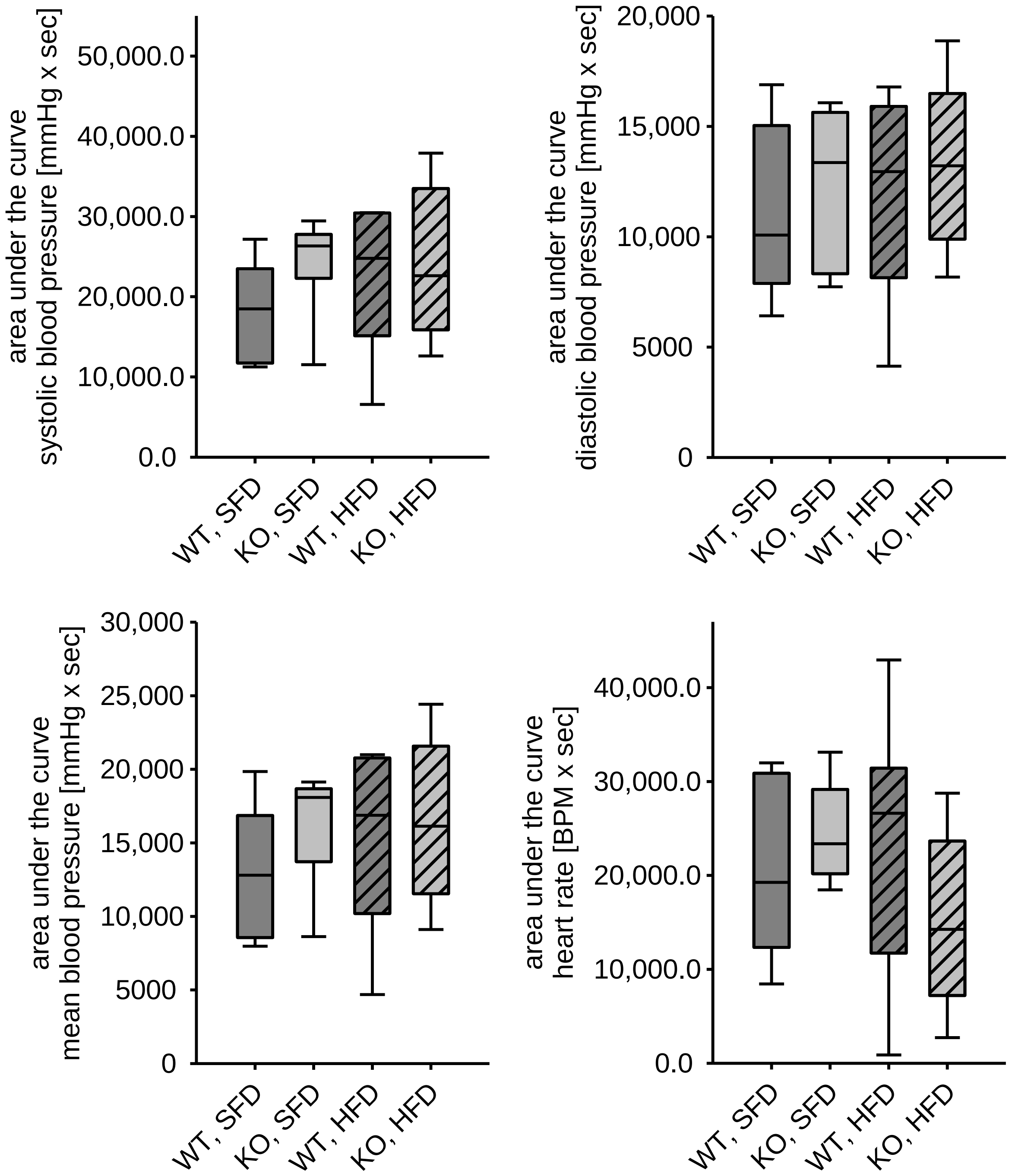

Figure S9. Area under the curve (AUC) of the vehicle-adjusted blood pressure change induced by angiotensin II in mice with (KO) or without (WT) deletion of the EGFR in EC. AUC was calculated with the trapezoid formula and measured until blood pressure or heart rate reached the initial volumes or maximum within 600 seconds after substance application. N(WT, SFD)= 6, N(KO, SFD)=6, N(WT, HFD)=7, N(KO, HFD)=12; Data given as mean ± SEM.

Supplementary Figure S10

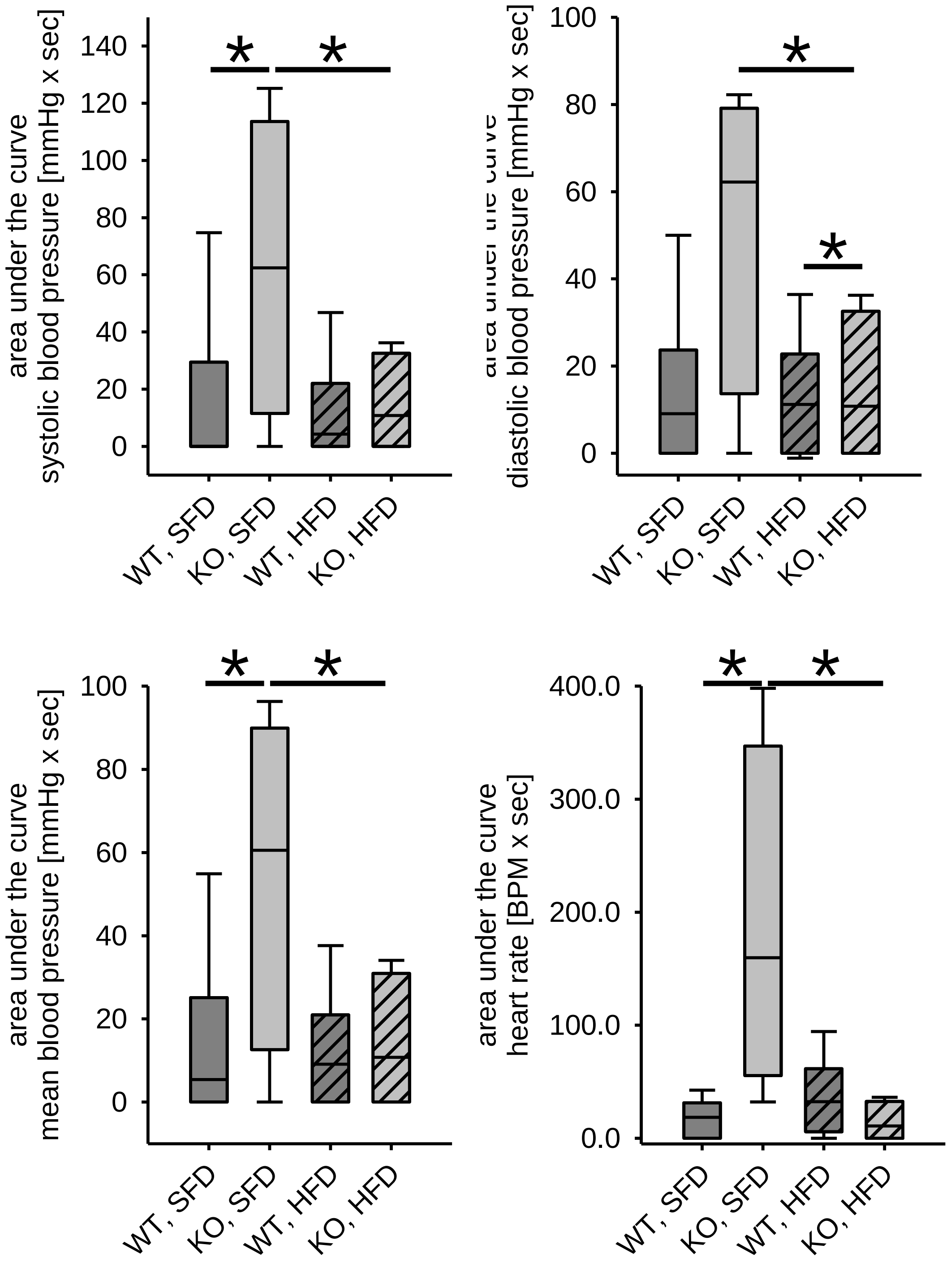

Figure S10. Area under the curve (AUC) of the vehicle-adjusted blood pressure change induced by angiotensin II in mice with (KO) or without (WT) deletion of the EGFR in VSMC. AUC was calculated with the trapezoid formula and measured until blood pressure or heart rate reached the initial volumes or maximum within 600 seconds after substance application. N(WT, SFD)= 9, N(KO, SFD)=4, N(WT, HFD)=13, N(KO, HFD)=4; Data given as mean ± SEM; * p ≤ 0.05 vs. respective control.
